# Characterization of *Monkeypox virus* infection in African rope squirrels (*Funisciurus sp*.)

**DOI:** 10.1371/journal.pntd.0005809

**Published:** 2017-08-21

**Authors:** Elizabeth A. Falendysz, Juan G. Lopera, Jeffrey B. Doty, Yoshinori Nakazawa, Colleen Crill, Faye Lorenzsonn, Lem’s N. Kalemba, Monica D. Ronderos, Andres Mejia, Jean M. Malekani, Kevin Karem, Darin S. Carroll, Jorge E. Osorio, Tonie E. Rocke

**Affiliations:** 1 US Geological Survey, National Wildlife Health Center, Madison, Wisconsin, United States of America; 2 Department of Pathobiological Sciences, School of Veterinary Medicine, University of Wisconsin, Madison, Wisconsin, United States of America; 3 Centers for Disease Control and Prevention, Atlanta, Georgia, United States of America; 4 University of Kinshasa, Kinshasa, Democratic Republic of Congo; 5 Animal Services (Pathology), Wisconsin National Primate Research Center, University of Wisconsin, Madison, Wisconsin, United States of America; NIAID Integrated Research Facility, UNITED STATES

## Abstract

Monkeypox (MPX) is a zoonotic disease endemic in Central and West Africa and is caused by *Monkeypox virus* (MPXV), the most virulent *Orthopoxvirus* affecting humans since the eradication of *Variola virus* (VARV). Many aspects of the MPXV transmission cycle, including the natural host of the virus, remain unknown. African rope squirrels (*Funisciurus spp*.) are considered potential reservoirs of MPXV, as serosurveillance data in Central Africa has confirmed the circulation of the virus in these rodent species [1,2]. In order to understand the tissue tropism and clinical signs associated with infection with MPXV in these species, wild-caught rope squirrels were experimentally infected via intranasal and intradermal exposure with a recombinant MPXV strain from Central Africa engineered to express the luciferase gene. After infection, we monitored viral replication and shedding via *in vivo* bioluminescent imaging, viral culture and real time PCR. MPXV infection in African rope squirrels caused mortality and moderate to severe morbidity, with clinical signs including pox lesions in the skin, eyes, mouth and nose, dyspnea, and profuse nasal discharge. Both intranasal and intradermal exposures induced high levels of viremia, fast systemic spread, and long periods of viral shedding. Shedding and luminescence peaked at day 6 post infection and was still detectable after 15 days. Interestingly, one sentinel animal, housed in the same room but in a separate cage, also developed severe MPX disease and was euthanized. This study indicates that MPXV causes significant pathology in African rope squirrels and infected rope squirrels shed large quantities of virus, supporting their role as a potential source of MPXV transmission to humans and other animals in endemic MPX regions.

## Author summary

*Monkeypox virus* (MPXV) is a virus closely related to *Variola virus*, the cause of smallpox, and it causes a similar, though less severe, disease in humans in Central and West Africa. This disease is particularly dangerous for children and people with compromised immune systems and the mortality rate is estimated to be about 10% in the more pathogenic Central African strains. Unlike *Variola virus*, MPXV is primarily transmitted to humans from animals, but it is not known which species commonly carry and spread this virus. Rope squirrels in the genus *Funisciurus* have previously been linked to the virus in Central Africa. In this study, rope squirrels were experimentally infected with MPXV and subsequently monitored for signs of disease, as well as the amount of virus they shed in their bodily fluids. The results of this study showed that rope squirrels became moderately ill but not all died. They shed large amounts of virus in oral, nasal, rectal, and ocular secretions. This information helps public health doctors and epidemiologists understand the potential risks of contacting rope squirrels for local populations in Africa and will help disease ecologists understand how MPXV is maintained and transmitted between animals and to humans.

## Introduction

*Monkeypox virus* (MPXV) is a zoonotic *Orthopoxvirus* (OPXV) endemic to West and Central Africa. MPXV causes monkeypox disease (MPX) in humans, characterized by fever, maculopapular rash, and lymphadenopathy [3], with a mortality rate of approximately 10% in the more pathogenic Central African strains of the virus [4]. Since the 1980’s, this disease has mostly been confined to Central Africa, although serological evidence suggests it may also circulate in West Africa [5,6]. Recent studies have suggested an increase in human cases in Central Africa, suggesting that this may be an emerging infectious disease [1,7]. The increasing incidence of MPX may be due to waning worldwide OPXV immunity. Smallpox vaccination campaigns, which also provided protection against MPXV, largely ceased in the 1980’s [7,8]. Future use of the smallpox vaccine for prevention of MPX is restricted by the large number of side effects of vaccination, especially in immunocompromised populations [9,10,11]. Humans become infected with MPXV through contact with infected animals or humans, and no effective treatment for MPX is available [1,12]. Therefore, the most feasible public health intervention to protect humans from MPX is to identify and avoid exposure to reservoir species [1].

Although MPX is a zoonosis, the identity of the reservoir host(s) remains unknown [4,13]; some evidence suggests native African rodents play a role [14]. The virus was first isolated from a captive cynomolgus macaque (*Macaca fascicularis)* in 1958 and identified in human cases in 1971 [15]. The first of only two isolates of MPXV from a wild animal was found in a moribund rope squirrel (*Funisciurus anerythrus*) in Zaire (now Democratic Republic of Congo (DRC) [16]. Shortly after, serological evidence for infection was found in rope squirrels, sun squirrels (*Heliosciurus rufobrachium*) and non-human primates in DRC, however seroprevalence was highest in rope squirrels (24.7%) [2]. Most recently, seropositive rope squirrels were found in the area of a human MPX outbreak in DRC [1]. The second isolation of MPXV from a wild animal occurred in Cote d’Ivoire in 2012, from a dead sooty mangabey (*Cercocebus atys*) [17]. In West Africa, evidence of OPXV infection has been found in several species: Gambian pouched rats (*Cricetomys gambianus*), African dormice (*Graphiurus sp*.), rope squirrels (*Funisciurus sp*.), sun squirrels (*Heliosciurus sp*.), and ground squirrels (*Xerus sp*.)[5]. An outbreak of MPX in the US in 2003 was associated with the importation of pouched rats, rope squirrels, and African dormice from Ghana [18]. Thus, there is evidence that African rope squirrels, and perhaps pouched rats, may be important for MPXV maintenance in nature. Understanding the pathology and viral shedding in potential reservoir species provides valuable information needed to estimate the risks for humans in contact with these species.

To date, very little experimental work with MPXV has been completed in suspected host species due to the logistical barriers of transporting live animals to institutions with BSL3 laboratories. Similarly, despite years of field surveillance, very few animals have been found to be clinically ill with MPXV infection, increasing the complexity of studying the natural history of the virus within free-living animals. Here, we use *in vivo* bioluminescent imaging (BLI) to study MPXV infection in laboratory and wild-caught animals [19,20,21], allowing us to characterize the distribution and amount of MPXV replication in live animals in real time. This approach has several advantages for studying the pathogenesis of MPXV in potential reservoir species [19]. First, BLI reduces the number of animals used, as serial sacrifice at successive time points is not needed [22,23,24,25,26,27,28]. Secondly, compared to inbred laboratory models, wild animals are more variable genetically and may demonstrate greater variation in response to infection. Following individual animals through time can illuminate these differences [29]. Finally, viral infection may occur in reservoir species without clinical signs, but BLI can detect viral replication, including in sites that are not commonly sampled during timed sacrifice studies, such as skin, lymph nodes, or ovaries [28,29].

## Materials and methods

### Ethics statement

This work was approved by the Animal Care and Use Committee of the National Wildlife Health Center, protocol number EP090616A6. All work with live animals meets the guidelines set forth in the Guide for the Care and Use of Animals and the Animal Welfare Act and regulations [30], and was performed under an assurance (A4492-01) from the Office of Laboratory Animal Welfare (OLAW) within the U.S. Public Health Service. Anesthesia was performed using isoflurane inhalant anesthetic, with or without injectable dexmedetomidine. Euthanasia was performed by CO2 asphyxiation, while under isoflurane anesthesia.

### Animals

Nine rope squirrels (*Funisciurus anerythrus*) were captured in Kinshasa and surrounding areas and were housed at the University of Kinshasa Biology Department for quarantine and testing. Animals were tested for the presence of OPX antibodies at the Congolese National Veterinary Laboratory, as described below. After confirmation that all rope squirrels were negative for OPX antibodies, they were examined, treated with topical fipronil (Frontline spray, Merial, Duluth, GA), and then exported to the United States (US) with permission of the Democratic Republic of Congo Ministry of Agriculture and Environment and the Centers for Disease Control and Prevention. After arrival in the US, rope squirrels were transported and housed at the USGS National Wildlife Health Center (NWHC, Madison, WI) in the select-agent registered ABSL3 animal facility. Rope squirrels were quarantined for one month, during which time they were treated with praziquantel (Wedgewood Pharmacy, Swedesboro, NJ) and two doses of selamectin (Zoetis, Florham Park, NJ) for external and internal parasites. For the infection study, all animals were individually housed in polysulfide rat cages with filter-topped lids inside a hepa- filtered cabinet. Following the infection study, 400 base pairs of the cytochrome b gene of all animals were sequenced as described in S1 Text. No sequences from *Funscisiurus spp*. were previously listed in GenBank, and these sequences have been deposited. Analysis of these sequences revealed polymorphisms, which are also described in S1 Text.

### Experimental infection

Animals were infected with Central African MPXV that expresses firefly luciferase (MPXV/Congo/Luc+). The production and comparison of this virus to wild type MPXV were previously described [31]. This recombinant virus was produced in the laboratory of Dr. Tonie Rocke at the National Wildlife Health Center and the original parental strain (MPXV-2003-Congo-358) was isolated in the Republic of Congo and kindly provided by Dr. Inger Damon at the Centers for Disease Control and Prevention. All work with recombinant *Monkeypox viruses* was approved by the NWHC Biosafety Committee. Four animals were infected intranasally (IN) by inoculating 5 μL in each nostril of phosphate buffered saline (PBS) containing 1 x 10^6^ plaque forming units (PFU) of MPXV/Congo/Luc+. Four animals were infected intradermally (ID) by placing 10 μL of 1 x 10^6^ PFUs of virus diluted in PBS onto a shaved area of skin in the dorsal midscapular area and then piercing into the dermis with a 26g needle 10 times, through the droplet. A sentinel animal received 10 μL of PBS IN and was housed in the same cabinet as the other squirrels. After infection, animals were observed twice daily for clinical signs.

### *In vivo* imaging

Squirrels were imaged and sampled on days 3, 6, 8, 11, 13, 15, 18, 22, 25, and 27 post infection (pi). Before *in vivo* imaging, animals were sedated with intramuscular dexmedetomidine (Zoetis, Florham Park, NJ) and anesthetized with isoflurane (Phoenix Pharmaceutical, St. Joseph, MO). After day 11 pi, dexmedetomidine was discontinued and anesthesia consisted of isoflurane alone. Animals were injected with 150 mg/kg of luciferase substrate (GoldBio, St. Louis, MO) intraperitoneally before being imaged using the IVIS 200 series *in vivo* imager (Perkin Elmer, Waltham, MA). Images were collected 30 minutes after injection (determined to be the time of peak luciferase distribution) using Living Image version 4.2 software (Perkin Elmer, Waltham, MA). Both dorsal and ventral images were collected, with two animals imaged together. Region of interest (ROI) analysis was performed with hand-drawn ROIs that encompassed the entire animal within the field of view. The tail was not visible in all views. Total luminescence was estimated as mean average radiance [p/s/cm^2^/sr] of ventral and dorsal views of individual animals and plotted with the time of infection [29].

### Sample collection

During anesthesia for imaging, animals were also weighed, examined for lesions, bled, and sterile polyester swabs were used to sample oral, nasal, rectal, and ocular mucosal surfaces. All swabs were placed into tubes containing 400 μL of Dulbecco’s Modified Eagle’s Medium (DMEM) supplemented with 1 μg/L amphotericin B, 100 U/mL penicillin, 100 μg/mL streptomycin, and 0.05 mg/mL gentamycin (Life Technologies, Grand Island, NY, USA). Blood was collected from the facial vein and placed into serum separator tubes (Sarstedt, Nümbrecht, Germany). Serum separator tubes were centrifuged at 2000 rpm for 10 minutes at room temperature. All swabs and sera samples were frozen at -80°C until later processing. Animals were euthanized if they lost more than 25% body weight, were unable to eat or drink, or had difficulty breathing. Euthanasia was performed by CO_2_ asphyxiation following isoflurane anesthesia. Carcasses were temporarily stored at 4°C and necropsied within 24 hours. Skin, tongue, superficial cervical lymph node, salivary gland, heart, lung, spleen, liver, intestine, bladder and ovary or testis tissue samples were collected and stored at -80°C until further processing. Sections of these tissues were also fixed in 10% formalin for histopathological analysis.

### Sample processing

Tissue pieces (25–60 mg) were homogenized in PBS, supplemented with 1% fetal bovine serum (FBS), using a Buller Blender Storm bead homogenizer with stainless steel beads (Next Advance, Averill Park, NY, USA). PBS with 1% FBS was added to the slurry to make a final concentration of 10% (wgt/vol). 200 μL of the tissue slurry was used for DNA extraction with the Zymo tissue g-DNA kit (Zymo Research, Irvine, CA). 200 μL of whole blood was used for DNA extraction with the Zymo g-DNA kit (Zymo Research, Irvine, CA). The quality of each DNA extraction was confirmed using a Nanodrop 2000 spectrophotometer (Nanodrop products, Wilmington, DE).

### Quantitative real-time PCR

Viral DNA in blood and tissues was quantified using real-time PCR to detect the E9L gene of orthopoxviruses using primers described by Li et al. [32]. SYBR Green PCR Master Mix (Applied Biosystems, Carlsbad, California, USA) was used in an iQ5 Real-Time PCR Detection System (Bio-Rad, Hercules, California, USA). DNA standards containing 4.15 ng to 4.15 x10^-7^ng were made by extracting total DNA from purified MPXV-Congo/Luc+ as described above, and creating eight 10-fold serial dilutions of the purified DNA in molecular grade water. Standards were aliquoted, and each aliquot was used no more than 5 times to prevent degradation of standards from repeated freeze-thaw cycles. Using these standards, assays were sensitive enough to detect approximately 2042.49 viral genomes in 0.1 mL. Standard curves, DNA concentration, and Ct value were calculated using the iQ5 Optical System Software, Version 2.1.97.1001 (Bio-Rad). The cutoff for fluorescence signal was determined by calculating the average and standard deviation of Ct values for standards across all PCRs. Samples with Ct values that were less than or equal to 2 standard deviations of the mean of the lowest concentration detectable within 40 cycles were considered positive. DNA quantities were used to calculate the number of viral genomes in the samples.

### Viral titration

Virus in swabs was detected and measured using a TCID_50_ assay and converted to PFU/mL as described elsewhere [21,29]. Briefly, 96 well-plates were seeded with Vero cells (ATCC CCL-81, Manassas, VA) to approximately 90% confluence. Swabs were sonicated and vortexed in the viral transport medium (described in sample collection) after thawing. Swab medium was diluted in DMEM with 1% FBS and antibiotics (1 μg/L amphotericin B, 100 U/mL penicillin, 100 μg/mL streptomycin, and 0.05 mg/mL gentamycin (Life Technologies, Grand Island, NY, USA)). 100 μL of eight 10-fold serial dilutions were added to each well in a 96 well plate. Plates were incubated at 37°C with 5% CO_2_ for 3 days before being fixed with 0.1% crystal violet in 10% formalin. After fixation and staining, viral plaques were counted and titer was determined with a Reed and Muench calculator [21,29].

### Antibody detection and serum neutralization assay

Antibodies for MPXV were detected by ELISA, using the methods outlined in Hutson *et al* [33]. Plates were coated with the lysate of *Vaccinia virus*-infected Vero cell lysate on one half and with lysate of uninfected Vero cells on the other half. Three serum samples were added in duplicate, to each side of the plate, in concentrations of 1:50 to 1:4800. Serum from a vaccinia-vaccinated human and a Gambian pouched rat that survived MPXV infection served as positive controls and the negative control was uninfected Gambian pouched rat serum from previous studies. A 1:20,000 dilution of the secondary antibody, an A/G conjugate was used, as well as SureBlue peroxidase substrate (KPL #52-00-01, Kirkegaard and Perry Laboratories, Washington DC, USA). After developing, plates were read at 450nm on a spectrophotometer (EL 800 Universal Microplate Reader, Bio-Tek Instruments Inc., Winooski, VT, USA). A cut-off value for each plate was calculated as two standard deviations above the mean of the optical density of the uninfected lysate side of the plate.

Pre-existing MPXV-specific antibodies were also detected by serum neutralization assay [21]. Briefly, Vero cells were cultured on 96-well plates. Cells were infected with 100 PFU per well of MPXV/Congo/Luc+. 100 μL of 6 serial 2-fold dilutions of rope squirrel serum (1:20, 1:40, 1:80, 1:160, 1:340, 1:680, and 1:1320) were added to the wells. After 24 hours, luciferase expression was detected using the Steadylite Plus luciferase detection kit (PerkinElmer, Waltham, MA) with the VICTOR Light 1420 plate luminometer (PerkinElmer, Waltham, MA). Inhibition of viral infection was detected by a 50% reduction in luminescence detection compared to infected cells without serum. Positive and negative controls were the same as for the ELISA, described above.

### Histopathological examination

Formalin-fixed tissues were sectioned and stained with routine hematoxylin and eosin (H&E) stains. Selected tissue sections were studied immunohistochemically by using an anti-vaccinia virus HRP rabbit polyclonal antibody (Thermo Fisher, Waltham, MA, USA) at a dilution of 1:200. The primary antibody was incubated with the section at 4°C overnight. A DAB substrate kit (Sigma Aldrich, St. Louis, MO) was used to detect the primary antibody, and Hematoxylin QS Vector (Vector Laboratories, Burlingame, CA) was used as a counterstain. Tissue sections from an uninfected rope squirrel were used as negative controls. H&E and IHC slides were reviewed by two pathologists (MR and AM).

### Statistics

Luminescence and shedding via oral, nasal, rectal, and ocular routes were assessed with a generalized linear model (log link function) using JMP 10 (SAS Institute Inc., 2012, Cary, NC), with days pi and route of infection (IN or ID) as the fixed effects.

## Results

### Clinical signs and mortality

All rope squirrels displayed moderate to severe morbidity following infection by either route. Mortality was 75% in the IN group and 50% ID group, although small group size prevented meaningful comparison of the mortality rate by route.

**Fig 1:**
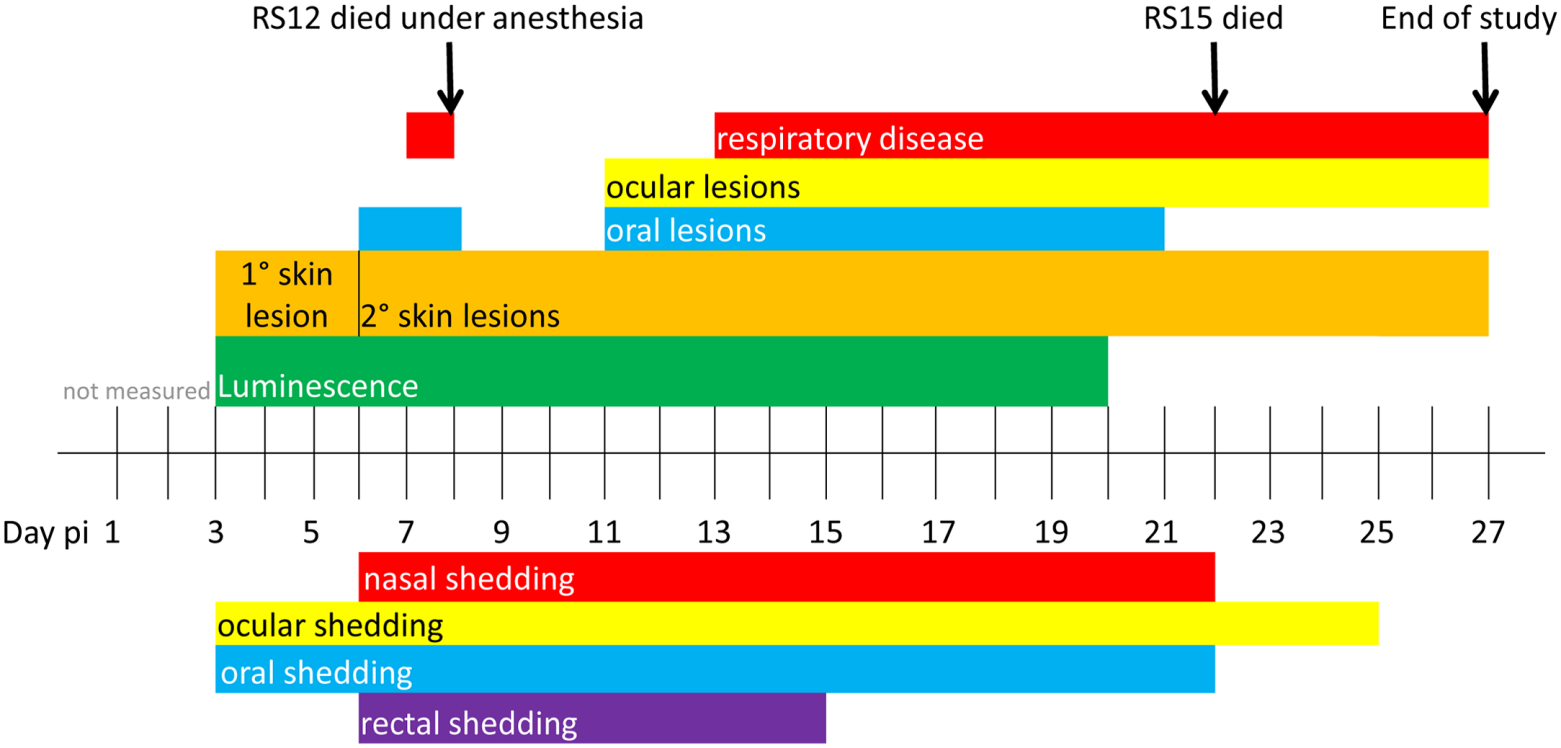
Progression of disease of rope squirrels infected intradermally with MPXV-Congo/Luc+. The days post infection (pi) of various clinical signs are shown for the group, above the time scale. The days of luminescence are shown in green. Days of shedding of virus in nasal, ocular, oral and rectal mucosal secretions, as detected by TCID_50_ assay, are shown below the time scale. Colors coordinate between clinical signs and route of shedding: nasal shedding and respiratory disease is shown in red. Ocular lesions and ocular shedding are shown in yellow. Oral lesions and oral shedding are shown in blue. Rectal shedding, for which no corresponding clinical observations were made, is shown in purple.

In the ID-infected group (RS12, RS15, RS17 and RS18), the primary skin lesions were visible beginning at day 3 pi. Typical poxviral lesions in the skin and in the oral cavity were evident by day 6 pi. One animal (RS12) died on day 8 pi during anesthesia, after one day of increased respiratory rate. Corneal lesions developed in one animal (RS17) on day 11 and both remaining animals displayed skin lesions, oral lesions, and nasal discharge. On day 22 pi, RS15 died. The remaining two squirrels (RS17 and RS18) survived the infection until the end of the study, day 27 pi, although skin lesions, respiratory disease, and ocular lesions were still present. Skin lesions progressed from papules, to pustules, then crusts, as described in other animal models such as prairie dogs and cynomolgus monkeys [34,35]. Fig 1 shows the disease progression of the ID group.

**Fig 2:**
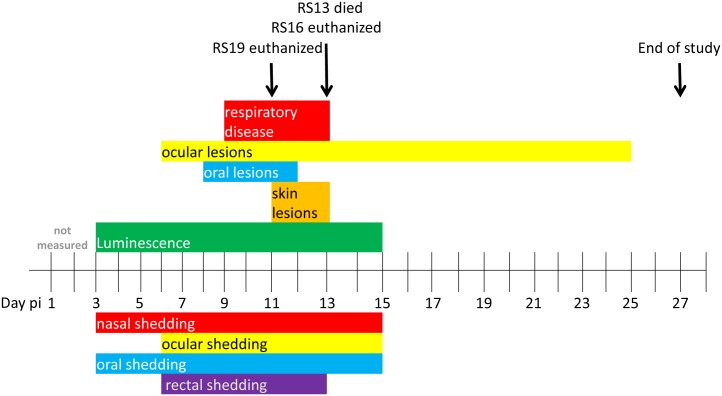
Disease progression and shedding of rope squirrels infected intranasally with MPXV-Congo/Luc+. The days post infection (pi) of various clinical signs are shown for the group, above the time scale. The days of luminescence are shown in green. Days of shedding of virus in nasal, ocular, oral and rectal mucosal secretions, as detected by TCID_50_ assay, are shown below the time scale. Colors coordinate between clinical signs and route of shedding: nasal shedding and respiratory disease is shown in red. Ocular lesions and ocular shedding are shown in yellow. Oral lesions and oral shedding are shown in blue. Rectal shedding, for which no corresponding clinical observations were made, is shown in purple.

In the IN-inoculated group (RS11, RS13, RS16, and RS19), ocular lesions appeared in one animal (RS13) on day 6 pi. Oral lesions were observed on day 8 pi, and three of four squirrels displayed severe respiratory disease beginning on day 9 pi with increased respiratory rate and nasal discharge. Some animals stopped breathing during anesthesia on several occasions and manual chest compressions were used to re-establish respirations. RS19 was euthanized on day 11 pi due to respiratory distress. Classic poxvirus type skin lesions, as described above, were visible in RS13 and RS16 between days 11 and 13 pi. On day 13, RS13 was found dead, and RS16 was euthanized immediately after imaging, due to respiratory distress. RS11 survived infection and the ocular lesions resolved in this animal by day 25 pi. Fig 2 shows the progression of disease of the IN group.

The sentinel animal, RS14, showed clinical signs of MPXV infection, including increased respirations, nasal discharge, and oral lesions beginning after day 13 pi. On day 18, it was euthanized due to respiratory distress, weight loss, and severe lethargy. A table describing the clinical signs in each individual animal is available in the supplementary material (S1 Table).

### Analysis of luciferase expression

**Fig 3:**
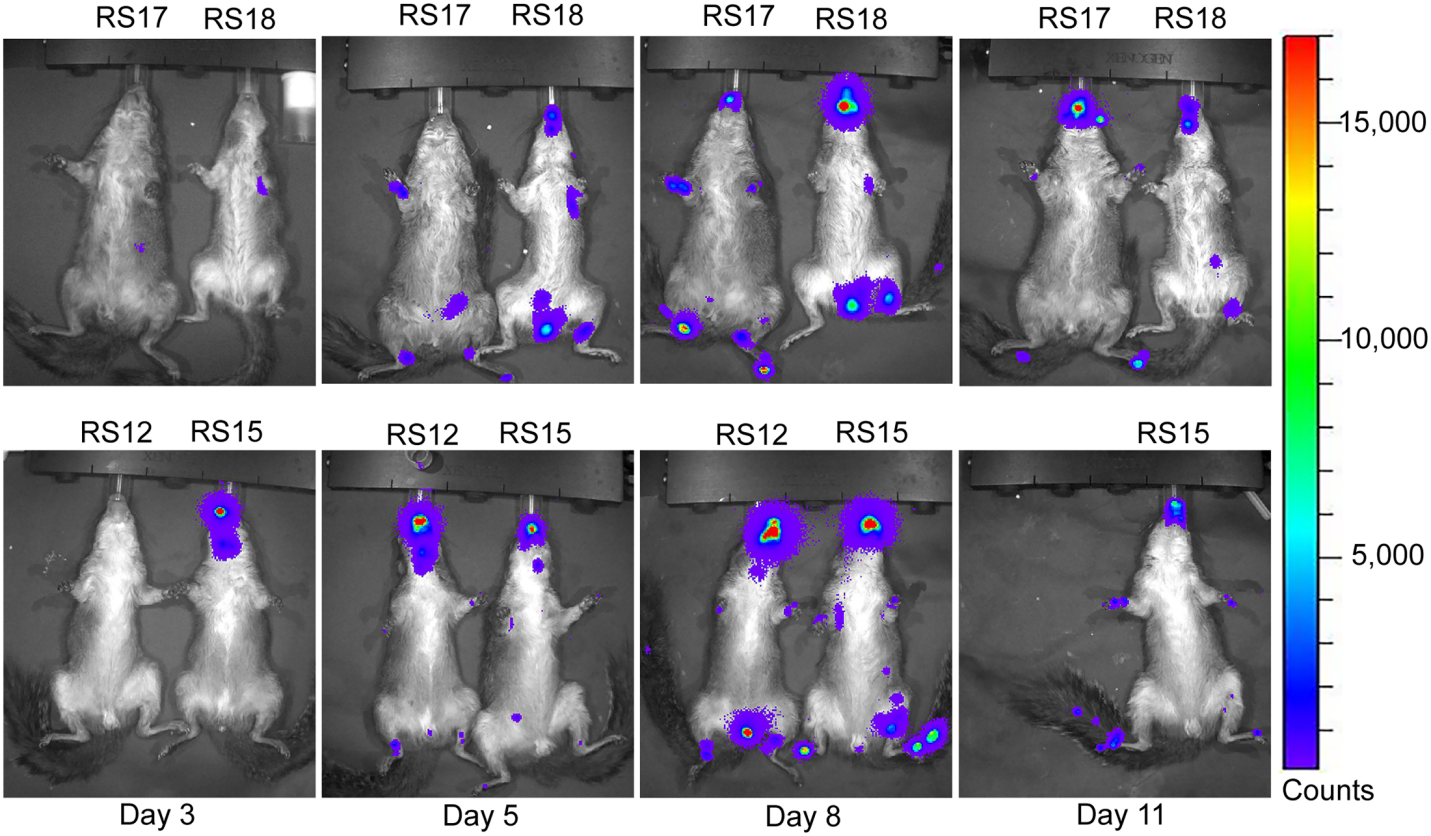
Ventral bioluminescent images of African rope squirrels intradermally infected with MPXV-Congo/Luc+ on days 3, 6, 8, and 11 post infection (pi). Color scale shows the intensity of luminescence in counts, with purple representing the lowest intensity, green the mid-range and red the highest intensity. Animals were infected on the dorsal scapular area. Luminescence in the oral and nasal areas, which begins on day 3 pi, is the result of replication of the virus distant from the initial site of infection. RS12 died on day 8 pi.

**Fig 4:**
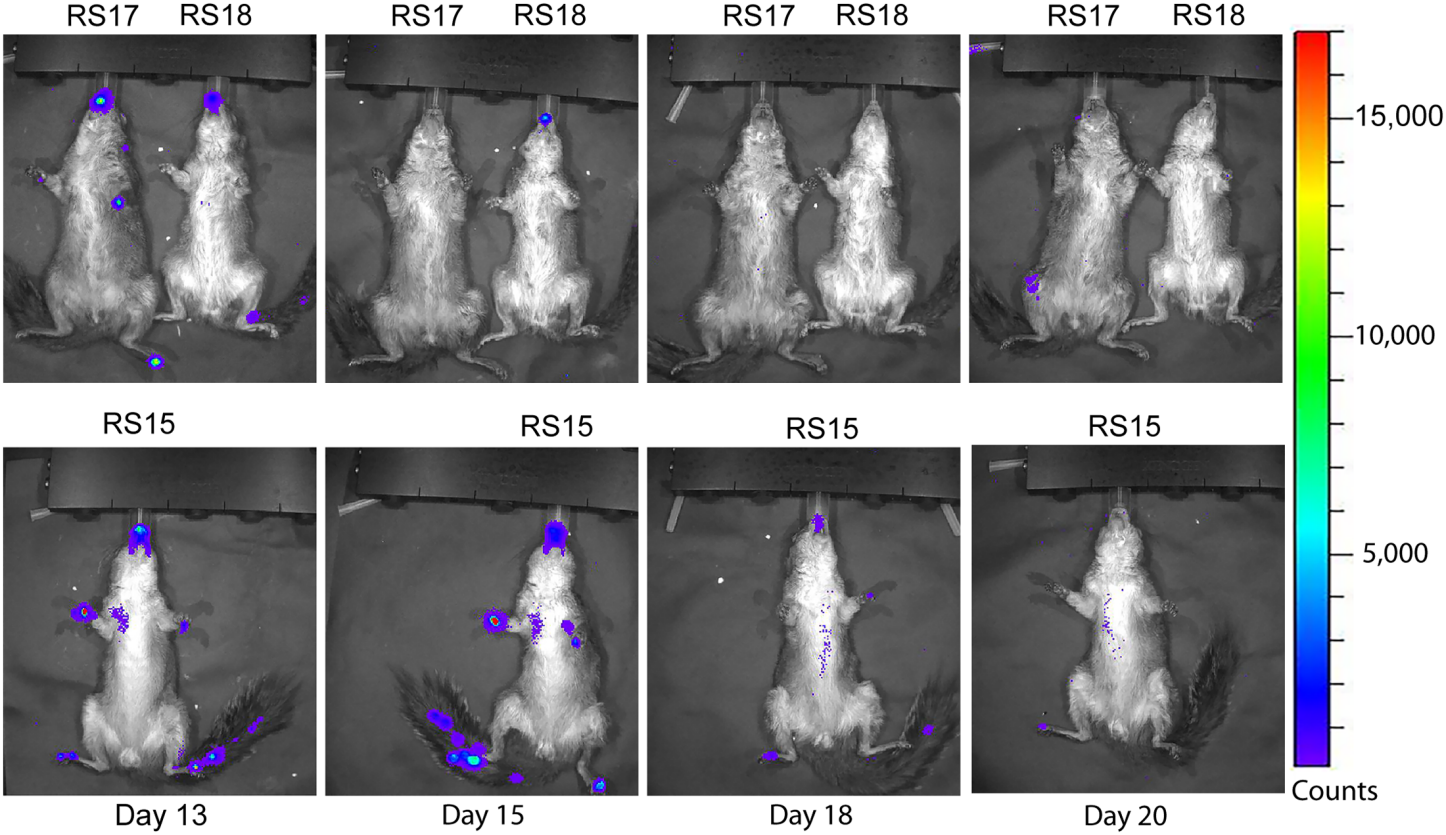
Ventral bioluminescent images of African rope squirrels intradermally infected with MPXV-Congo/Luc+ on days 13, 15, 18, and 20 post infection (pi). Color scale shows the intensity of luminescence, in counts, with purple representing the lowest intensity, green the mid-range and red the highest intensity. Luminescence, indicative of viral replication, in the oral and nasal areas continued until day 18 pi. Distal limbs and the tail were common sites of replication. These sites often corresponded to lesions on the feet.

**Fig 5:**
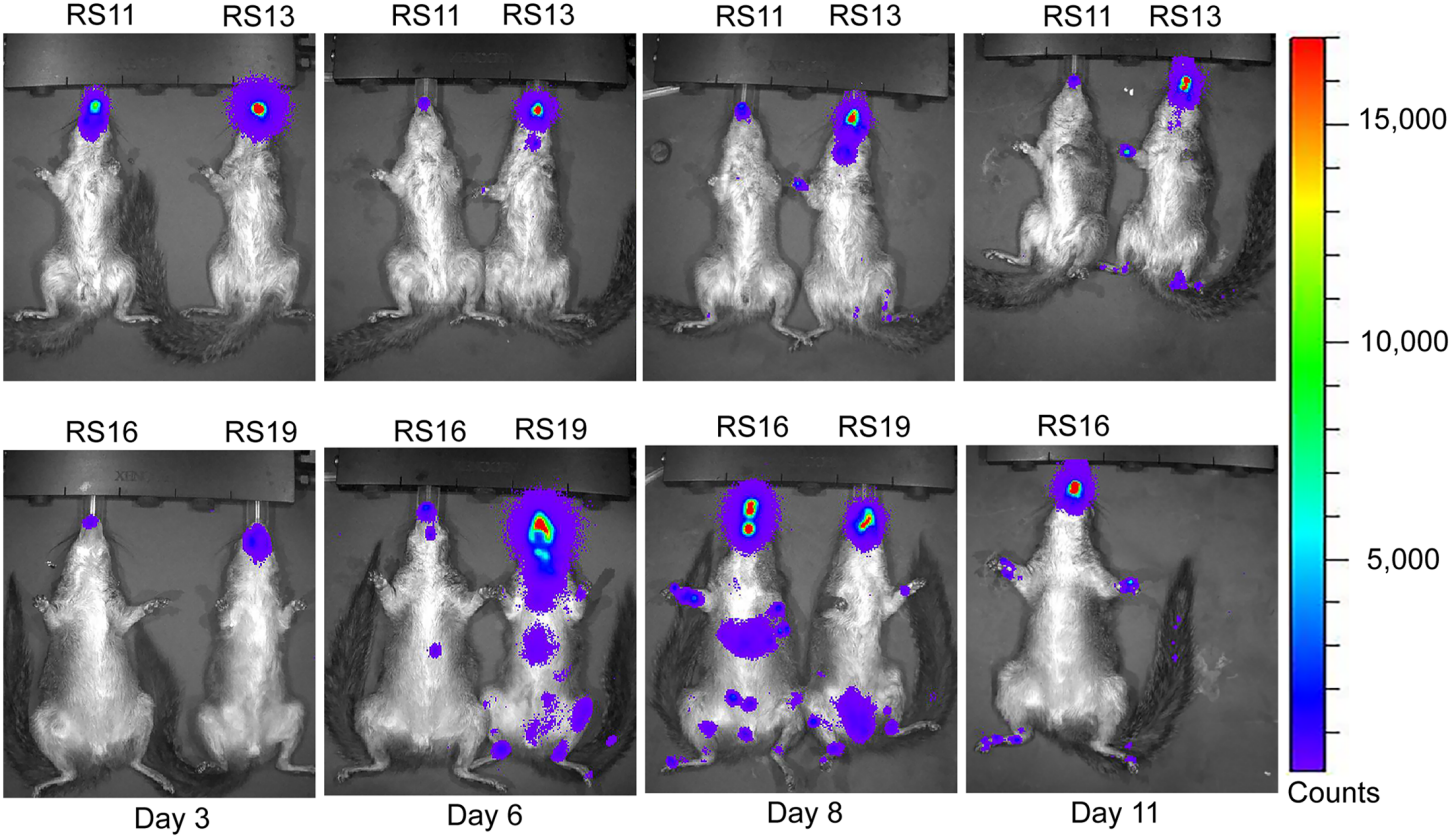
Ventral bioluminescent images of African rope squirrels intranasally infected with MPXV-Congo/Luc+ on days 3, 6, 8, and 11 post infection (pi). Color scale shows the intensity of luminescence, with purple representing the lowest intensity, green the mid-range and red the highest intensity. Detectable luminescence is present in the oral and nasal areas in all animals on day 3 pi, which was the site of inoculation in this group. By day 6 pi viral replication is detectable at distal sites. After day 11 pi RS19 was euthanized.

**Fig 6:**
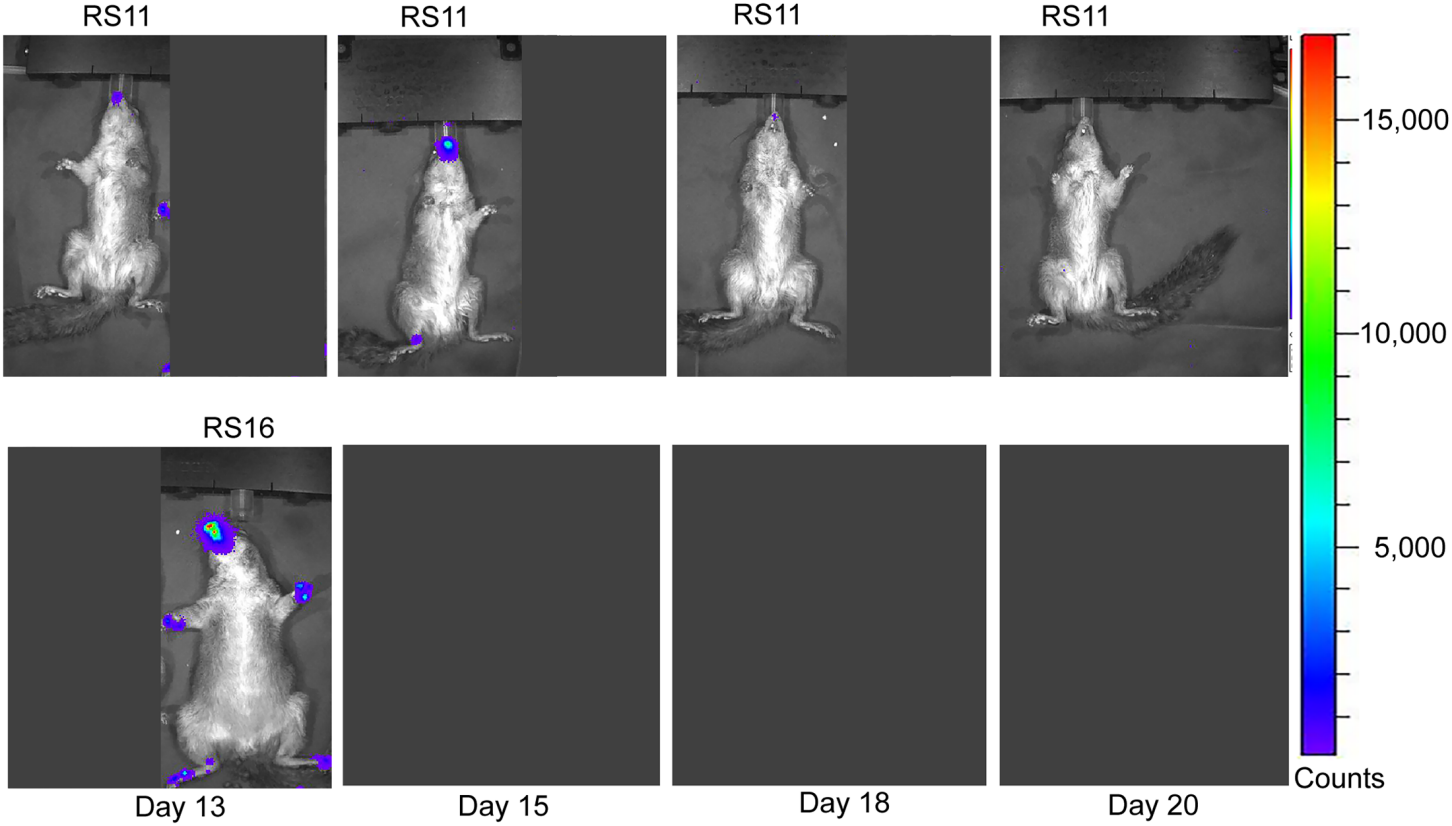
Ventral bioluminescent images of African rope squirrels intranasally infected with MPXV-Congo/Luc+ on days 13, 15, 18, and 20 post infection (pi). Color scale shows the intensity of luminescence, with purple representing the lowest intensity, green the mid-range and red the highest intensity. After day 13 pi, RS13 was found dead. RS16 was euthanized after imaging on day 13 pi. RS11 had a large amount of luminescence in the oral and nasal areas on day 13 pi, but decreased throughout the remainder of the days. By day 20 pi, luminescence is not visible in bioluminescent overlay images.

**Fig 7:**
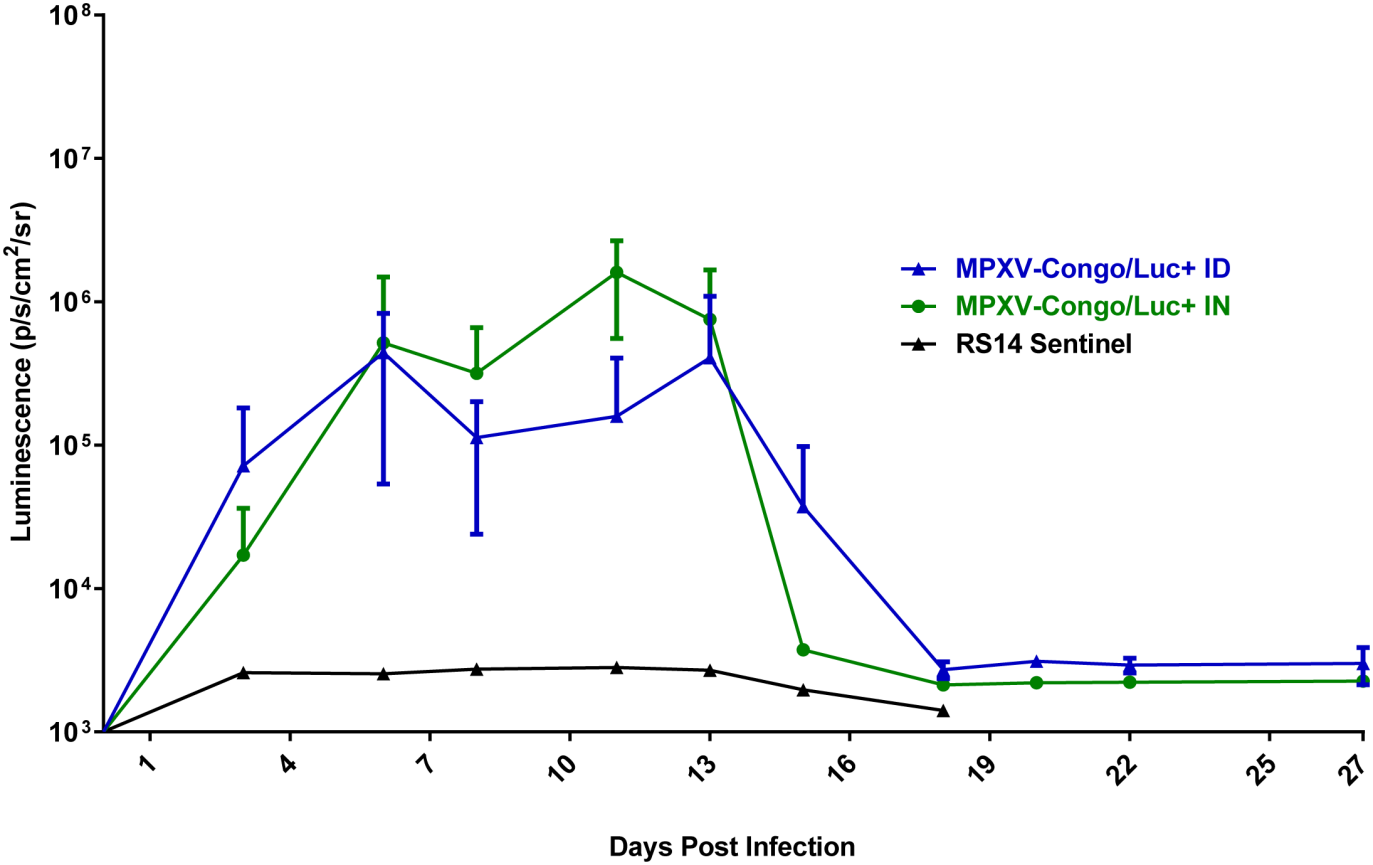
Quantification of luminescence of African rope squirrels infected intradermally (ID) and intranasally (IN) with MPXV-Congo/Luc+. Both the IN and ID groups showed increasing luminescence from days 0 to 6 post infection (pi), which decreased slightly on day 8 pi, then increased again. The IN group showed peak luminescence on day 11 pi, and the ID group showed peak luminescence on day 13 pi. For both groups luminescence was detected up to day18 pi, and then remained at the background level for the remainder of the study. The sentinel animal did have luminescence above background levels.

Luminescence overlays from the ventral view are shown for animals in the ID group in Figs 3 and 4, and for the IN group in Figs 5 and 6. Luminescence, indicative of viral replication, was present in the oral and nasal areas of both IN and ID groups by day 3 pi, the first time point of BLI (Figs 3 and 5). In the ID group, this location represents a secondary site of replication. The site of inoculation on the dorsal scapular area also had strong luminescence on days 3 to 11 pi (see data in S2 Table). In the ID group, luminescence peaked at day 6 pi and a second peak occurred on day 13 pi (Fig 7). In this group, the luminescence dropped down to background levels by day 20 pi (Figs 4 and 7). In the IN group, luminescence was visible in sites distal to the inoculation on day 6 pi (Fig 5). Luminescence increased more gradually and peaked on day 12 pi (Fig 7). After day 13 pi, three of four animals (75%) in the IN group had died or were euthanized, and the luminescence of the remaining animal, RS11, quickly decreased to background levels by day 18 pi (Figs 6 and 7). Overall, luminescence was slightly higher (P = 0.02) in the IN group than the ID group. The sentinel animal did not increase above background luminescence levels (Fig 7), which was surprising. Analysis of the virus infecting the sentinel animal proved to be a non-recombinant virus of the same parental strain. Follow-up studies on the MPXV/Congo/luc stocks discovered that they were incompletely purified and non-recombinant virus was present. Details of this analysis and conclusions are described in S4.

### Viral shedding

**Fig 8:**
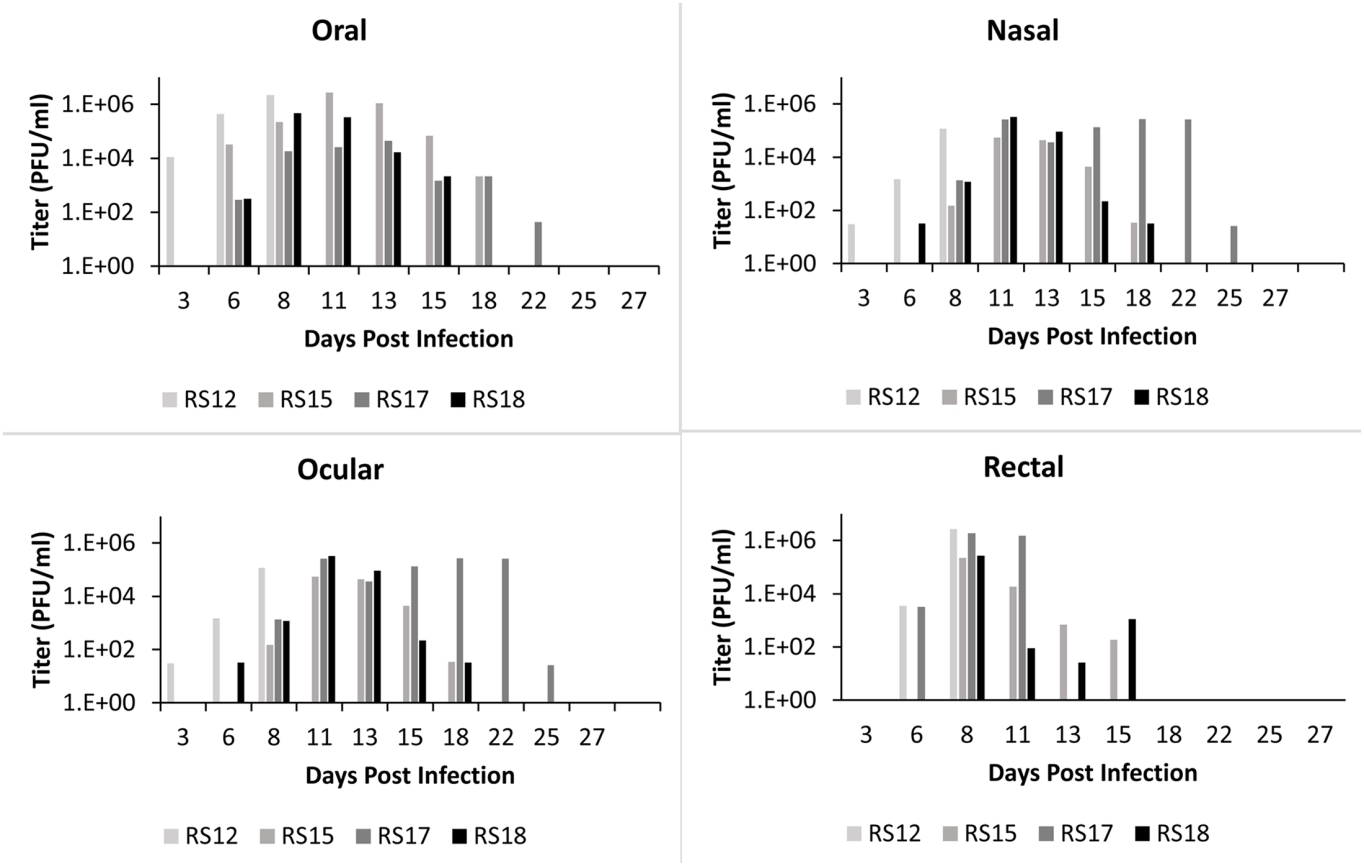
Shedding of MPXV in African rope squirrels infected intradermally with MPXV-Congo/Luc+. Swabs were taken from oral, ocular, nasal and rectal mucosa then tested by TCID_50_ assay to determine the concentration of virus shed in PFU/mL. Titers increased following infection, peaked between days 8 and 13, and then decreased for all animals except RS17, which showed consistently high titer from ocular swabs until day 25 pi.

**Fig 9:**
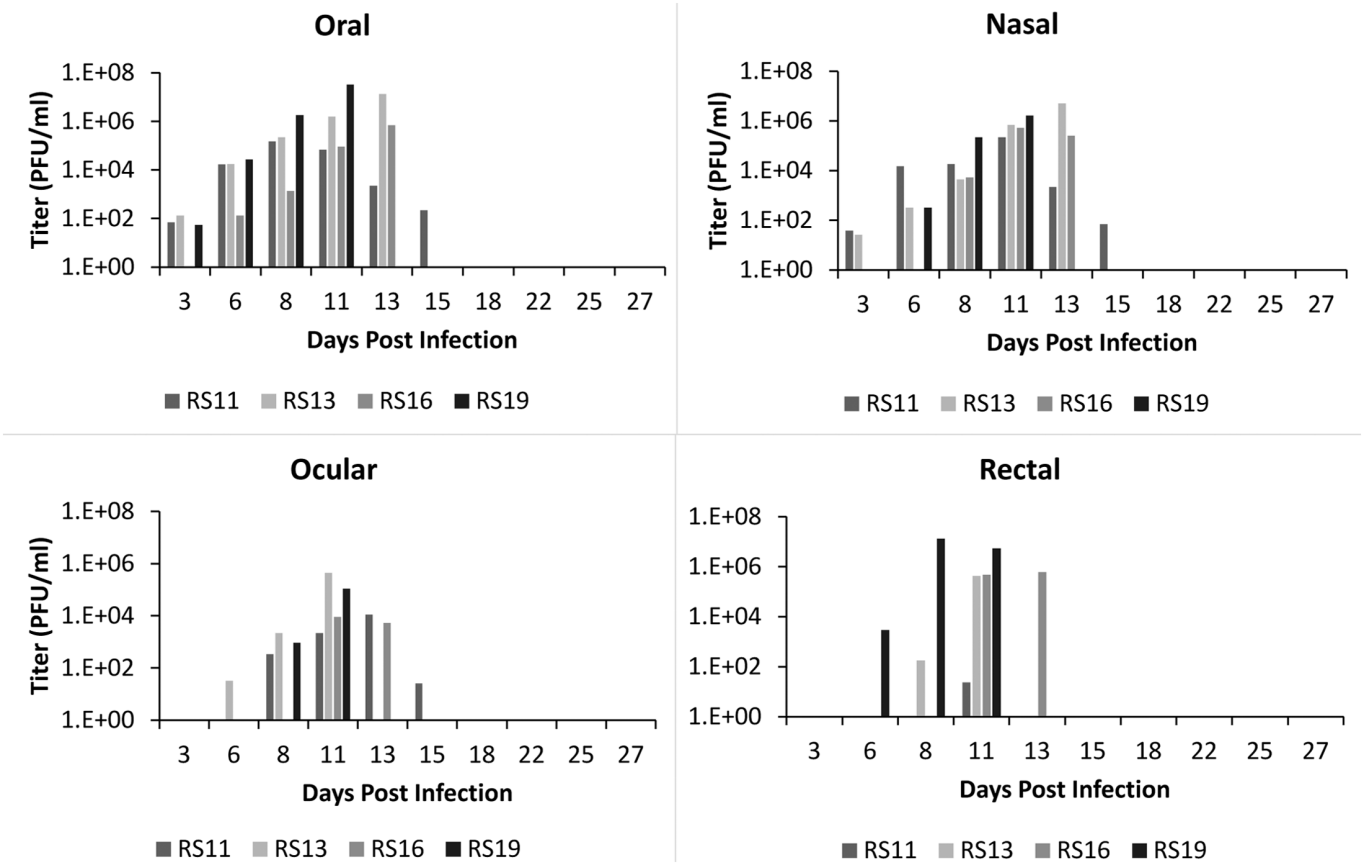
Shedding of MPXV in African rope squirrels infected intranasally with MPXV-Congo/Luc+. Swabs were taken from oral, ocular, nasal and rectal mucosa then tested by TCID_50_ assay to determine the concentration of virus shed in PFU/mL. Viral shedding increased following infection, peaked between days 8 and 13 pi, and then decreased for all animals. All viral shedding had ceased by day 15 pi in RS11, the lone survivor. All other animals in this group had died by day 13 pi.

**Fig 10:**
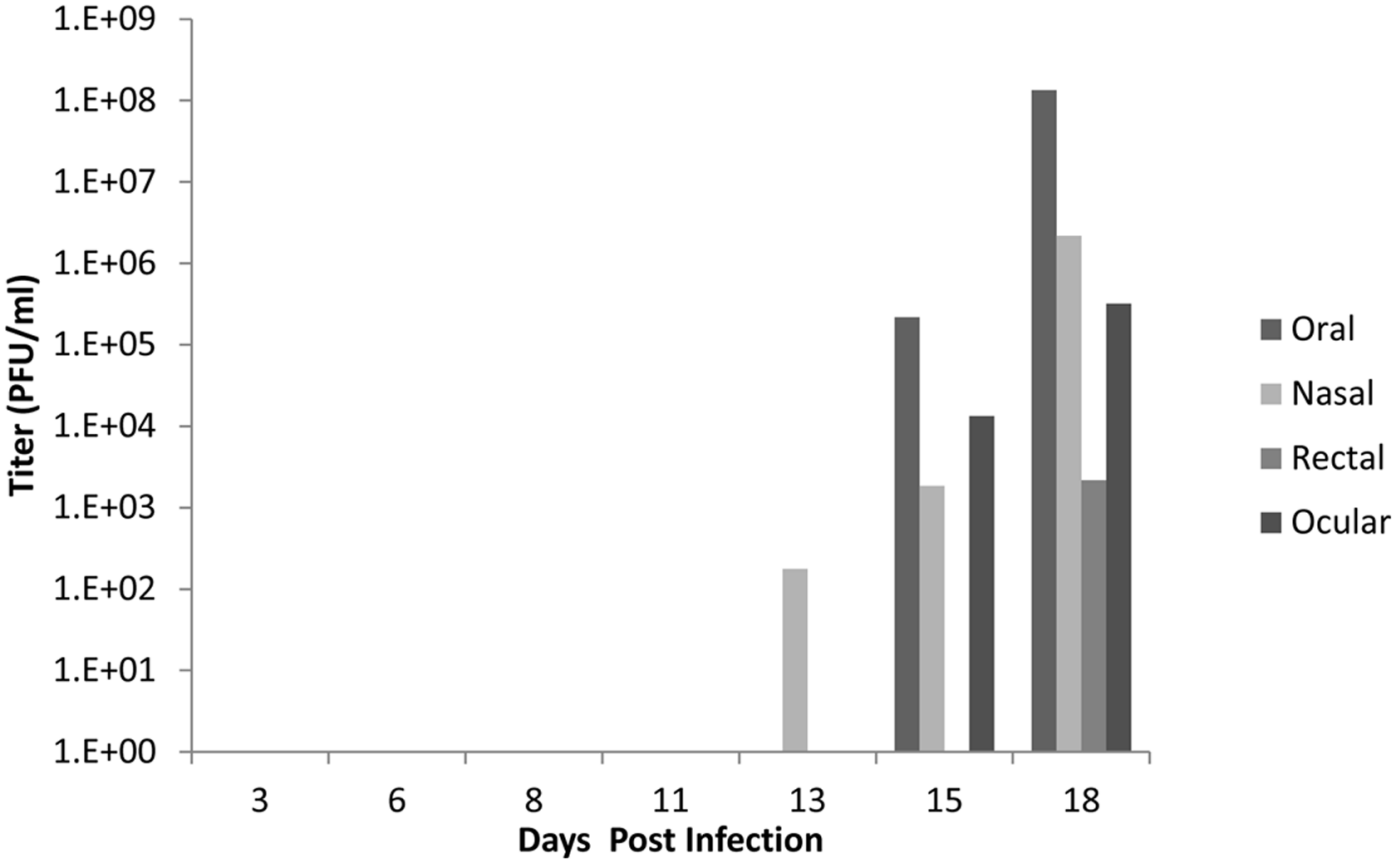
Shedding of MPXV by the sentinel African rope squirrel, RS14, following mock intranasal infection. The animal was housed in its own cage in the same room as the study animals, and was sampled and imaged following the same schedule as the other study animals. Swabs were taken from oral, ocular, nasal and rectal mucosa then tested by TCID_50_ assay to determine the concentration of virus shed in PFU/mL. Titer for all swabs increased from day 13 pi (oral) or day 15 pi (nasal, ocular, rectal) until day 18 pi, when the animal was euthanized. Oral shedding was the highest, with a final titer greater than 1.3 x 10^8^ PFU/mL on day 18 pi.

All infected animals in both ID and IN groups shed high amounts of virus (Figs 8 and 9). The highest concentration of virus was detected in oral secretions. For the ID group, shedding peaked on days 8–11 pi (Fig 8). For the IN group, the highest shedding was detected on days 11–13 pi (Fig 9). For both groups, shedding decreased after day 13, although one animal (RS17) had persistent ocular lesions and shed high amounts of virus in ocular secretions. Animals shed live MPXV as long as day 25 pi and as early as day 3 pi, even before the onset of clinical signs. Shedding via any route (oral, nasal, rectal, or ocular) was not significantly different (P>0.05) via route of infection (ID or IN). Although there was no luminescence detected from the sentinel (RS14), it did shed live MPXV (Fig 10) beginning on day 13 pi, consistent with the appearance of clinical signs around that time, and continuing until the time of euthanasia. The virus isolated was confirmed as MPXV by the MPXV-specific primers described by Li et.al. [32]. The lack of luminescence of this isolate was confirmed by the plate luminescence assay (S2 Text). PCR was also used to confirm that the MPXV isolated from this animal was from the inoculum used to infect the other animals; however it did not contain any of the GPT/luc cassette (S2 Text).

### Viremia and tissue titers estimated by quantitative PCR

The first whole blood sample was collected on day 8 pi. Viremia, as estimated by real-time PCR detection of viral genomes, is shown in Table 1. Viremia was detected after day 8 pi in three of four animals in each group. All samples from RS12, which were of small volume, were used for serology. The detection of viremia by real time PCR seems to show a cyclical pattern of viremia, although true viremia was not confirmed by culture of live virus from blood. RS14, the sentinel, had detectable viremia on days 15 and 18 pi, although clinical signs and viral shedding were already present by day 13 pi. The pattern of viral distribution in tissues was highly variable (Table 2). The highest number of viral genomes was detected in primary skin lesions (ID group only), lips, and tongues (IN and ID groups). Many of the tissues of the three surviving animals (RS11, RS17, and RS18) were negative, although each of them contained viral DNA in one or more tissues at the end of the study.

**Table 1:** Viremia as estimated by real-time PCR in rope squirrels. Quantities shown are shown in genomes/0.1 mL. RS14 was a mock-infected sentinel animal. Viremia was detected starting at day 15 pi, although clinical signs and viral shedding were detected earlier. RS15 had a prolonged viremia and it was detected when it died on day 22 post infection, although many of the clinical signs were apparently reduced by this time.

	Intranasal				Intradermal				Sentinel
Day	RS11	RS13	RS16	RS19	RS15	RS17	RS18	RS12	RS14
**8**	2.82E+04	9.82E+04	1.85E+04	N/S	6.90E+04	4.22E+04	2.65E+03	N/S (D)	Neg
**11**	N/S	N/S	N/S	1.88E+06 (E)	N/S	N/S	N/S		N/S
**13**	N/S	N/S (D)	4.37E+05 (E)		N/S	N/S	N/S		N/S
**15**	6.87E+03				5.84E+04	7.62E+03	3.06E+03		3.70E+04
**18**	N/S				N/S	N/S	N/S		3.10E+05 (E)
**22**	Neg				1.54E+04 (D)	Neg	Neg		
**27**	Neg (S)					Neg (S)	Neg (S)		

S = survived, D = died, E = euthanized, N/S = no sample, Neg = negative

**Table 2:** Viral DNA in tissue of African rope squirrels infected intranasally and intradermally with MPXV-Congo/Luc+. Tissue samples were collected after death or euthanasia. Data in the table represents the number of genomes per mg of tissue, as determined by quantitative real time PCR. Tissues that were not available for certain animals are represented by “N/S” for no sample. Letters near each animal number specify whether or not the animal lived through the course of the study.

	Intranasal				Intradermal				NC
Tissues	RS11 (S)	RS13 (D)	RS16 (E)	RS19 (E)	RS15 (D)	RS17 (S)	RS18 (S)	RS12 (D)	RS14 (E)
**Skin Lesion**	Neg	Neg	Neg	Neg	7.77E+03	Neg	Neg	Neg	1.61E+08
**Primary Lesion**	N/S	N/S	N/S	N/S	2.42E+06	2.07E+06	236.1*	8.46E+07	N/S
**Lip**	Neg	9.84E+06	3.30E+06	1.83E+07	Neg	247.2*	N/S	4.30E+07	Neg
**Reproductive Organs**	Neg	Neg	Neg	1.67E+06	Neg	Neg	Neg	Neg	3.68E+04
**Eyelid**	Neg	Neg	Neg	1.18E+06	Neg	Neg	Neg	Neg	Neg
**Tongue**	Neg	6.85E+05	6.46E+04	Neg	Neg	Neg	Neg	6.94E+06	1.02E+07
**Stomach**	Neg	Neg	Neg	Neg	Neg	Neg	Neg	1.48E+05	2.08E+05
**Lung**	Neg	3.30E+04	2.08E+03	6.76E+04	5.34E+02	Neg	Neg	1.67E+04	4.90E+04
**Submandibular Lymph Node**	Neg	1.96E+03	Neg	5.59E+04	Neg	Neg	Neg	2.35E+05	Neg
**Bladder**	Neg	9.71E+03	Neg	1.08E+03	Neg	Neg	Neg	Neg	Neg
**Small Intestine**	Neg	Neg	Neg	Neg	Neg	Neg	Neg	Neg	2.42E+04
**Spleen**	Neg	Neg	7.37E+02	1.03E+04	Neg	Neg	Neg	3.28E+03	2.41E+04
**Kidney**	Neg	Neg	Neg	1.42E+03	Neg	Neg	Neg	Neg	1.12E+04
**Salivary**	Neg	Neg	7.18E+02	Neg	4.30E+02	Neg	Neg	2.10E+03	Neg
**Brain**	Neg	Neg	7.22E+02	4.08E+02	Neg	Neg	Neg	Neg	1.89E+03
**Large Intestine**	1.18E+05	Neg	Neg	Neg	299.2*	Neg	Neg	Neg	6.55E+02
**Liver**	Neg	Neg	Neg	3.89E+02	Neg	Neg	Neg	Neg	2.93E+04
**Heart**	Neg	Neg	3.11E+02	Neg	Neg	Neg	Neg	Neg	1.61E+03

S = survived, D = died, E = euthanized, Neg = negative

* Ct values lower than lowest positive standard

### Measurement of antibody responses

**Fig 11:**
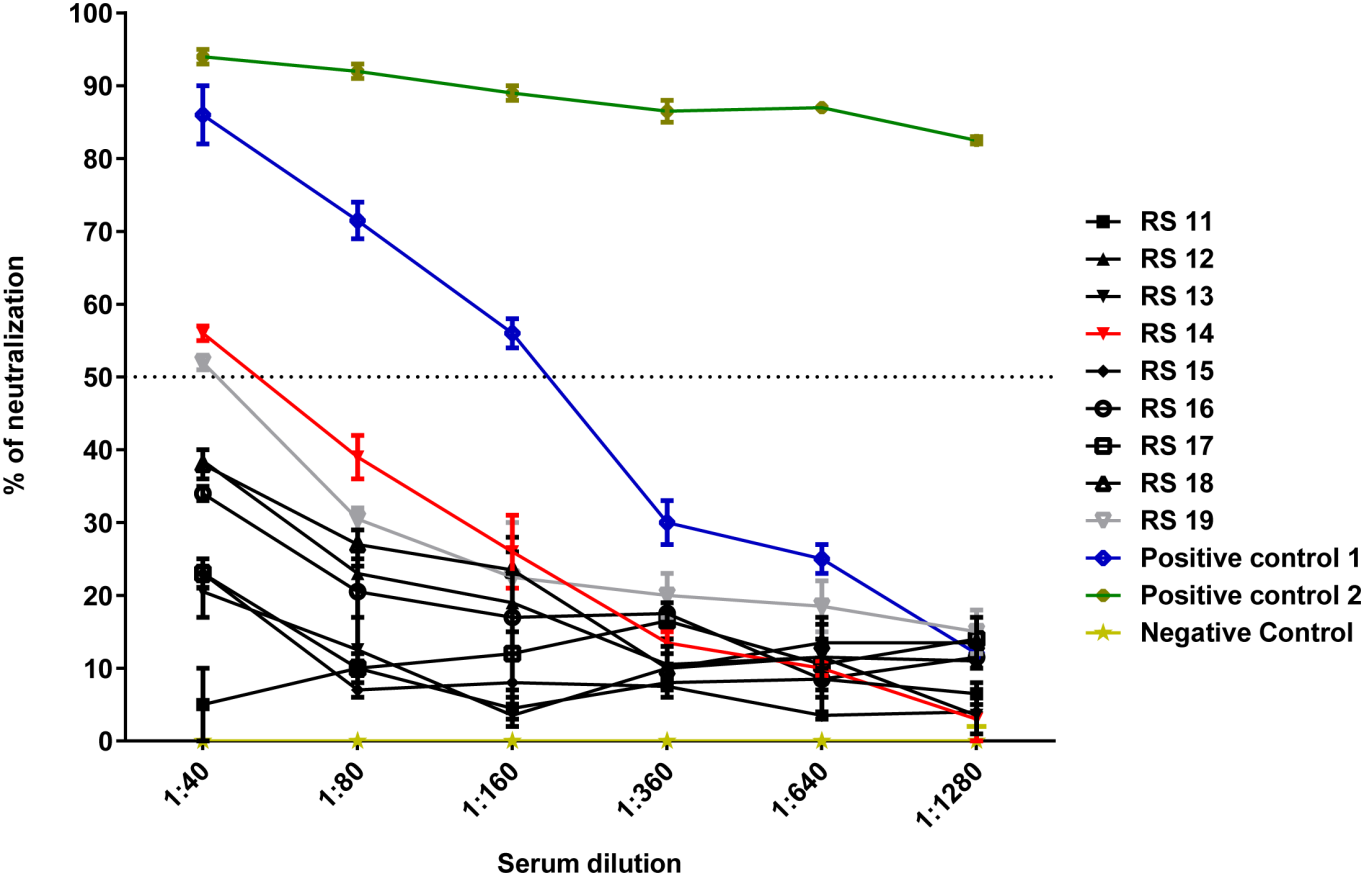
Pre-infection neutralizing activity of serum of rope squirrels against MPXV/Congo/Luc+. Positive controls were serum from a human vaccinated with vaccinia smallpox vaccine (positive control 1) and serum from a Gambian pouched rat (*Cricetomys gambianus*) that survived MPXV infection (positive control 2). Serum from an uninfected Gambian pouched rat was used as a negative control. RS14 and RS19 had a slightly greater than 50% reduction in viral luminescence at 1:40 dilution. However, this was not considered a protective titer. Neither of these animals survived infection with MPXV.

**Fig 12:**
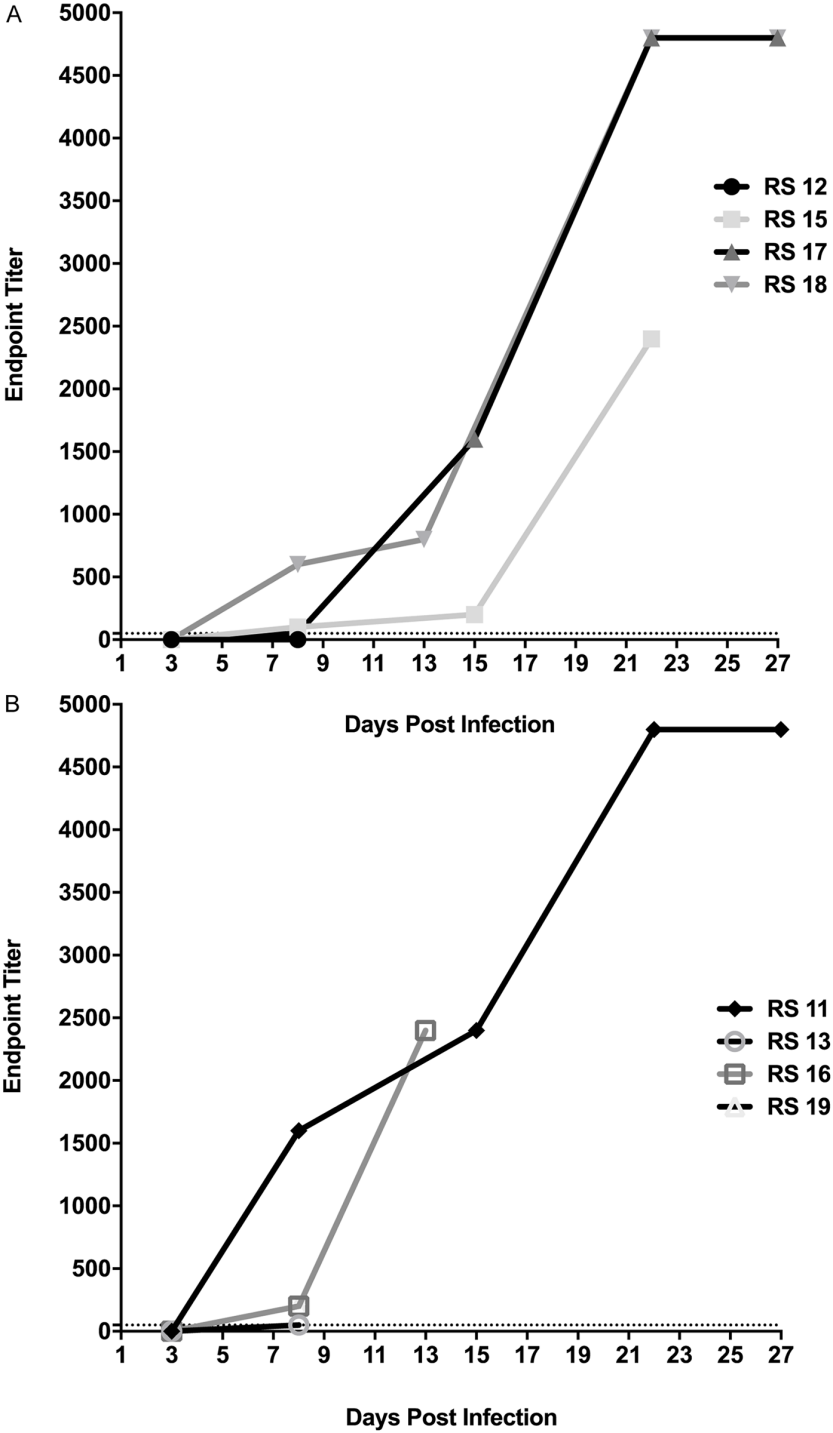
Endpoint ELISA titers of African rope squirrels infected intradermally (A) and intranasally (B) with MPXV-Congo/Luc+. Antibodies increased beginning on day 6 post infection (pi) and reached 1:4800 (the highest dilution tested) in the three surviving animals (RS17, RS18, and RS11). RS15 and RS16 had antibody titers of 1:2400 on the day they died, days 22 and 13 pi respectively.

All animals were sero-negative prior to the study, as measured by serum neutralization assays (Fig 11). Serum from RS14 and RS19 diluted to 1:40 displayed a slight reduction in viral neutralization. This was a high concentration of the serum and was not considered a positive anti-MPXV antibody titer. Antibody titers, as measured by ELISA, began to rise around day 6 pi (Fig 12). RS12, RS19, and RS13 did not have detectable antibody titers on the days of their death (days 8, 11, and 13 pi, respectively). RS15 and RS16 had titers of 1:2400 upon their death on days 22 and 13, respectively. The surviving animals in both groups had titers of 1:4800, the highest dilution tested.

### Histopathologic and immunohistochemical analysis

Histopathologically, typical MPXV infection lesions were present in the skin, the oral cavity, and the lungs. Table 3 shows the distribution and frequency of microscopic lesions. In the ID-infected group (RS12, RS15, RS17 and RS18), cutaneous lesions were characterized by epithelial hyperplasia and necrosis, most prominently in the stratum basal and deep stratum spinosum. In two animals (RS17 and RS15), small foci of epithelial hyperplasia were evident with formation of intraepithelial pustules and multiple dermal foci of lymphoplasmacytic inflammation that was predominantly perivascular. Cutaneous lesions were severe in RS12 and RS15 characterized by epidermal spongiosis, acanthosis, acantholysis, and occasional cell necrosis, predominantly in the basal layer, leading to ulceration. Superficial dermal inflammation was composed of neutrophils, macrophages, and necrotic debris. Surrounding vessels exhibited reactive endothelium and walls with edema, fibrin (fibrinoid change), and acute inflammatory infiltrates (RS12). In contrast, in the sections of the IN-infected group (RS13, RS16 and RS19), cutaneous lesions were characterized only by epithelial hyperplasia and multiple dermal foci of perivascular lymphoplasmacytic inflammation. Cutaneous lesions were not evident in the skin sections collected from RS11.

**Table 3:** Frequency and distribution of histologic lesions in African rope squirrels infected intradermally (ID) or intranasally (IN) with MPXV-Congo/Luc+. (+) indicates a lesion was found in that individual animal within that tissue. (−) indicates that no lesion was seen in the sections collected and examined microscopically. The frequency of the lesion was calculated for all 9 animals, regardless of route of infection.

	ID Infection				IN Infection				Sentinel	
Tissues	RS11	RS13	RS16	RS19	RS15	RS17	RS18	RS12	RS14	Frequency
**SkinLesion**	-	+	+	+	+	+	+	+	-	77% (7/9)
**Lip**	-	-	+	-	-	-	-	-	-	11% (1/9)
**Tongue**	-	+	+	-	-	-	-	-	+	33% (3/9)
**Lung**	+	+	+	+	+	_+_	+	+	+	100% (9/9)
Lymph Node*	-	+	-	-	-	-	-	+	-	22% (2/9)
**Spleen**	-	+	-	-	+	+	-	-	-	33% (3/9)
**Kidney**	-	-	+	+	-	+	+	+	+	66% (6/9)
**Heart**	-	-	+	-	+	+	-	-	-	33% (3/9)

*Submandibular

Lesions in the oral cavity were only present in RS13, RS16 and RS14. Oral epithelium, including tongue and labial mucosa, were similar morphologically to those in the skin and were characterized by epithelial hyperplasia, intracellular edema (ballooning degeneration), necrosis and ulceration with mixed inflammation in the adjacent submucosa. Scattered and poorly defined eosinophilic intracytoplasmic inclusion bodies were observed in epidermal and oral epithelium.

In the respiratory tract, all animals except RS11 exhibited mild lymphoplasmacytic interstitial pneumonia with hyperplasia of the peribronchial associated lymphoid tissue. The alveolar septae were mildly thickened and peripheral alveoli were often flooded with edema and foamy alveolar macrophages. RS15, RS12, RS14 and RS19 also presented moderate to severe alveolar histiocytosis with presence of numerous multinucleated giant cells.

In addition to epidermal and pulmonary lesions that are typical in other species infected with MPXV, renal lesions were common in this study. Many animals (RS12, RS14 RS16, RS17, RS18 and RS19) presented widespread renal tubular degeneration and multiple foci of perivascular lymphoid and plasmacytic inflammation in cortex and renal pelvis. Lesions in the heart included multifocal lymphoid and plasmacytic pericarditis, myocarditis and endocarditis, observed in RS15, RS16, RS17 and RS18. Atrium sections exhibited multiple areas of perivascular lymphoplasmacytic infiltrate, as well as in the adjacent epicardial adipose tissue. Lymphocytic gastritis and esophagitis were observed in RS15 and RS14. Lastly, a focus of necrotizing lymphadenitis of the superficial cervical lymph node occurred in one animal (RS12), accompanied by proliferation of fibroblasts and macrophages.

No lesions attributable to MPXV were seen in liver, spleen, urinary bladder, mammary gland, parathyroid gland, adrenal gland, reproductive organs, small and large intestine, gallbladder, pancreas or brain. Immunohistochemically, MPXV antigen was only detected in skin and tongue lesions. There were areas corresponding to the epithelial lesions with numerous intralesional cells (stratum basale and spinosum epithelial cells) with strong positive cytoplasmic staining. Positive immunostaining in the skin was observed in the sentinel animal, RS14. No positive staining was observed in control sections.

## Discussion

Rope squirrels have been linked epidemiologically to several outbreaks of human monkeypox [1,18], but little was previously known about how the virus replicates and is shed in this species. All rope squirrels in this study displayed clinical evidence of monkeypox disease after infection and shed high amount of virus by all routes of shedding tested. Viral shedding appeared to be independent of the route of infection. In cynomolgus macaques, the LD50 of MPXV in aerosol route is estimated at 7.8 x10^4^ pfu [36]. In the highly susceptible prairie dog model, the LD50 of MPXV by the IN route is estimated at 5.9 x 10^3^ pfu [37]. The rope squirrels in this study shed up to 1.34 x 10^7^ pfu/ml, indicating that only a few microliters of nasal discharge or saliva from an infected rope squirrel could cause lethal infection in susceptible hosts.

Although MPXV lead to the death of the majority of animals in this study, they shed infectious virus for 5–22 days. Mortality was 50% and 75% in the ID and IN groups, respectively. All infected animals were quite lethargic and had some degree of respiratory compromise. In fact, the morbidity associated with this disease may actually augment transmission of the virus, as they could make easier prey items for animals or humans.

In comparison to another suspected MPXV reservoir, the Gambian pouched rat, rope squirrels have higher mortality and higher morbidity. In a study using the same dose and routes, 33% of Gambian pouched rats died and 100% became sick after ID infection, but no mortality or morbidity following IN infection [19]. In fact, after direct infection by the ID route, the majority of the animals experienced mild symptoms. These animals also shed infectious virus by oral, nasal, ocular and rectal routes, with peak shedding being 1.85 x 10^6^ pfu/ml [19]. Another recent study of MPXV infection in Gambian pouched rats demonstrated mild clinical disease after ID infection with only 4x10^4^ pfu and shedding as high as 1.64 x 10^7^pfu/ml [38], similar to shedding reported here in rope squirrels. Although it is still unknown whether either or both species are MPXV reservoirs, it appears that the Gambian pouched rat may be less susceptible than rope squirrels to clinical disease caused by MPXV.

The pathology of MPXV in rope squirrels shared many similarities to that in other species. Lesions of the skin were classic pox lesions. Likewise, oral lesions were very similar to those in other species, including prairie dogs, Gambian pouched rats, and humans [4,19,20]. Lymphoplasmacytic interstitial pneumonia was also similar to that seen in other rodents [20,21,39]. Unlike other sciurids, such as ground squirrels and prairie dogs, the rope squirrels did not have hepatic or splenic lesions [39,40]. A large majority of the rope squirrels did have renal tubular degeneration with perivascular lymphoid and plasmacytic inflammation [41]. Renal lesions have not been reported in other species. Immunohistochemistry only detected orthopoxvirus antigen in skin and oral cavity. Based upon the presence of giant cells within the lung in conjunction with the lymphoplasmacytic pneumonia, it seems likely that the virus was present in the lung, but below the IHC sensitivity threshold. OPX antigen was also not detected in the kidney or heart, despite renal and cardiac lesions. Although no other causes were evident by special stains, a non-MPX cause of these lesions cannot be ruled out. Myocarditis has been reported in humans vaccinated with vaccinia virus [42], though is not reported in human cases of MPX [4]. Endocarditis has been reported in prairie dogs following MPXV infection, although viral antigen was also not detected [20]. Viral DNA was detected in the kidneys of some animals in this study and has been reported in other animal studies [18,20,39], although none of these studies reported histological lesions in the kidney.

An interesting, but unexpected event that occurred during this study was the infection of the sentinel animal, potentially through exposure to aerosols. The animal was housed individually in a hepa-filtered cage but within the same cabinet as the infected animals. However, the sentinel animal could have been exposed to aerosols from infected animals while it recovered from anesthesia, because the hepa-filtered lid was open to allow for observation and other animals were being handled and anesthetized during this time. All infected animals displayed respiratory disease and were possibly creating highly infectious aerosols in close proximity to the sentinel animal. Fomite contamination was also possible but probably less likely, because this animal was handled first during every sampling day, 10% bleach was used to disinfect handling gloves between animals, and Maxima detergent and 70% ethanol were used to clean the anesthesia tubing and nose cones between animals, as well as the surfaces of the imager and exam table. This suggests the possibility that MPXV is easily transmitted by aerosols to rope squirrels. Future studies should be designed to determine the effectiveness of this route of transmission in rope squirrels.

The infection of the sentinel animal was also surprising because the isolated virus matched the parent type virus but was not luminescent. This was confirmed by a plate luminometer with several samples from RS14, as well as by confirming absence of the luciferase (luc) gene in the viral genome. Sequencing of this isolate confirmed it was the parental virus used to create the MPXV-Congo/luc construct and further analysis of the stock virus inoculum detected both luciferase expressing and wild-type MPXV-Congo. A full description of assays and results described above is found in S2 Text. The finding of wild-type virus in the MPXV-Congo/luc stocks limits the interpretation of some of our data. Many replicates of cell culture assays have demonstrated a relationship between viral titer and luciferase expression for this MPXV construct [31]. This relationship is proportional, based on the number of recombinant viruses present in the volume tested. All experimentally infected squirrels in this study were inoculated with the same volume, at the same time, from the same vial of virus, so the mixture of recombinant and non-recombinant viruses should be the same for all. Therefore it is reasonable to compare the luminescence of animals experimentally infected in this study. Further, growth curves in cell culture did not detect an advantage of the wild-type virus over the recombinant virus and tissue tropism and replication kinetics were not different between MPXV-Congo and MPXV-Congo/luc viruses in a mouse model [31]. However, luminescence cannot be used to detect differences in viral expression between a secondarily infected animal and directly inoculated animals, because the quantity of recombinant vs. parental virus that infected this animal cannot be known. Also, *in vivo* imaging cannot be used in this case to determine the entire distribution of MPXV within an animal. In previous studies in prairie dogs, recombinant luciferase-producing virus was found in areas that were not detected by *in vivo* imaging [19,20]. This was attributed to light attenuation from overlying tissues in large animals, and this may be true, but the presence of some non-luciferase-expressing virions likely also contributed to this decreased sensitivity. Future studies using the guanine phosphoribosyltransferase (gpt) selection method for production of recombinant poxviruses will be followed by additional rounds of plaque purification (five were used to create these stocks), and testing for wild-type virus by specifically designed PCR assays.

Despite the incomplete purification of the MPXV-Congo/luc stocks, our observations of MPX infection in rope squirrels demonstrate that they have the potential to play a role in the epidemiology of MPXV in Central Africa. Evidence that MPX is circulating in rope squirrels in Central Africa is increasing, although their role in human infection and MPX epidemiology remains unclear. Rope squirrels have been found to be serologically positive in several studies [1,2,18]. Only 2 wild isolations of MPXV have been made from animals, one of which was a rope squirrel [16]. However, in a recent outbreak of MPXV in DRC, no association was found between contact with rope squirrels and human infection [1]. Additionally, a recent survey of 34 villages in the Tshuapa region of DRC did not detect contact with a rope squirrel carcass in the previous 30 days [43]. Perhaps, humans are unlikely to contact infected rope squirrels. Another possible role of rope squirrels is as an amplifying host for the virus, increasing the likelihood of transmission to other species more likely to be contacted by humans, such as monkeys or Gambian pouched rats [43]. The data presented here shows that rope squirrels shed high amounts of virus by several routes, thereby supporting the potential of this species to amplify the virus and infect other animals. The high morbidity caused by MPX infection in rope squirrels could lead to increased interactions with predators, including monkeys. Also, as poxviruses are known to be quite environmentally stable [41], rope squirrels could serve to contaminate the environment through fecal and oral shedding.

### Conclusions

In conclusion, rope squirrels are very susceptible to MPXV infection, displaying high morbidity and moderate mortality. During clinical infection, rope squirrels shed large quantities of virus, which supports their potential role in the epidemiology of MPXV in Central Africa.

## Supporting information

S1 Text Mitochondrial cytochrome b sequencing of rope squirrels (*Funisciurus anerythrus*). (PDF)

S2 Text Analysis of luciferase cassette in MPXV/Luc+. (PDF)

S1 Table Progression of disease in individual rope squirrels (*Funisciurus anerythrus*) infected with MPXV-Congo/Luc+. (XLSX)

S2 Table Mean average radiance [p/s/cm^2^/sr] of ventral and dorsal views of individual rope squirrels (*Funisciurus anerythrus*) infected with MPXV-Congo/Luc+. (PDF)
