# Supplementary material for: Characterization of Monkeypox virus infection in African rope squirrels (Funisciurus sp.)
Source: PLoS Negl Trop Dis. 2017 Aug 21;11(8):e0005809. doi: 10.1371/journal.pntd.0005809 (PMC5578676; doi:10.1371/journal.pntd.0005809)
Supplement: S1 Text — (PDF) [file pntd.0005809.s001.pdf]

S1 Text. Mitochondrial cytochrome b sequencing of rope squirrels (*Funisciurus anerythrus*)

Materials and Methods

DNA was extracted from 100-200 ul of whole blood from each rope squirrel. 400 base pairs of the mitochondrial cytochrome b (Cyt b) gene was amplified and sequenced. Primers were 5' CGA AGC TTG ATA TGA AAA ACC ATC GTT G 3' (MVZ05) and 5' GCC CTC AGA AGG ATA TTG TCC TCA TGG 3' (400R) [39]. PCR products were purified using Zymo PCR purification kit (Zymo Research, Irvine, CA) and the amplication concentration was calculated using a Nanodrop 2000 spectrophotometer (Nanodrop, Wilmington, DE). Sanger sequencing was performed at the University of Wisconsin Biotechnology Center (Madison, WI). DNA sequences were edited and aligned using Geneious 8.0.5 software. Phylogenetic trees were constructed using a neighbor-joining algorithm (Kimmura and 1,000 bootstraps) in MEGAN5 software [40]. BLAST search in non-redundant databases were performed and sequences were submitted to NCBI GeneBank. The GeneBank AY371098.1 and AY371103.1 were used as outgroups in phylogenetic analyses.

Results and Discussion

In the BLAST search, no rope squirrel (*Funisciurus* sp.) matching sequences were encountered. The closest related sequences belonged to the particolored flying squirrel (*Hylopetes alboniger*). The phylogenetic reconstruction showed that two main branches were supported (bootstraps = 100) (Fig. A). The experimental infection groups were distributed between the two branches. These are the first sequences reported in the genus *Funisciurus*. The distance of the branches and number of nucleotide substitutions suggests that the study population displayed high genetic

diversity. This may have impacted the diversity of pathological outcomes found in the infection trial. However, morbidity and mortality occurred within both genetic groups.

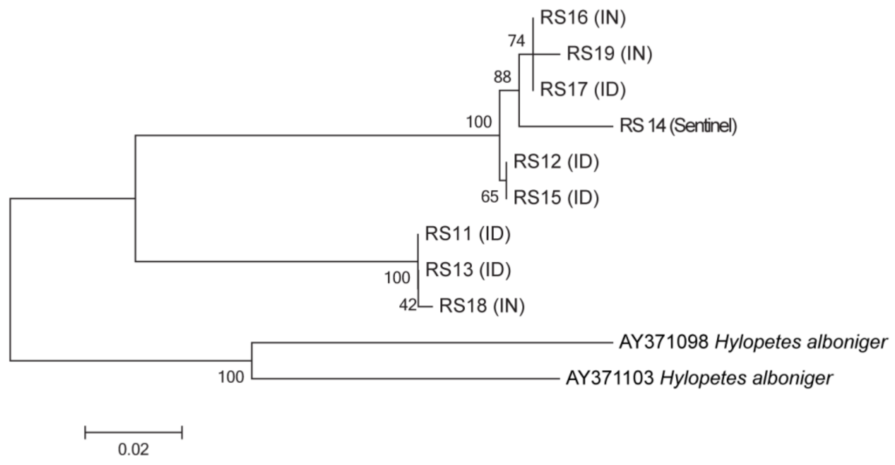

Figure A. The phylogenetic tree, constructed based on 400 bp sequences of the cytochrome b gene, show that there are two different genetic groups within the group of rope squirrels, (*Funisciurus anerythrus*) infected with Monkeypox virus. The infection groups are indicated after the individual animal numbers, as either intradermal (ID), intranasal (IN), or sentinel.
