# Supplementary material for: Characterization of Monkeypox virus infection in African rope squirrels (Funisciurus sp.)
Source: PLoS Negl Trop Dis. 2017 Aug 21;11(8):e0005809. doi: 10.1371/journal.pntd.0005809 (PMC5578676; doi:10.1371/journal.pntd.0005809)
Supplement: S2 Text — (PDF) [file pntd.0005809.s002.pdf]

**S2 Text. Analysis of luciferase cassette in MPXV/Luc+**

## Introduction

During the experimental infection trial RS14, the sentinel animal, began to show clinical signs consistent with monkeypox infection, including lethargy, nasal discharge, and pustular lesions on the tongue. Increased luminescence was not detected in this animal at any time point. Histopathological analysis of these lesions was similar to experimentally infected animals and several tissues and swabs were positive by real time PCR (tongue, liver, oral swabs, and blood).

## Materials and Methods

To evaluate the cause of the lack of luciferase expression by the MPXV that infected the sentinel animal, we isolated virus from tongue and liver, which were highly positive for MPXV real time PCR. Luciferase expression of these viruses was measured using a plate luminometer. Vero cells were cultured on 96-well plates. Cells were infected with four ten-fold dilutions of tissue slurry in DMEM. 100 PFU of MPXV/Congo/Luc+ was used as a positive control. At 24 and 48 hours post-infection, luciferase expression was detected using the Steadylite Plus$^{TM}$ luciferase detection kit (PerkinElmer, Waltham, MA) with the VICTOR Light 1420 plate luminometer (PerkinElmer, Waltham, MA). A duplicate plate was made above was made and fixed at 48 hours post-infection with 1% crystal violet in 10% formalin.

To evaluate the potential loss of the luciferase cassette by mutation or presence of a different strain MPXV, we performed PCR using 10 sets of primers (Table A) that cover the cassette and flanking regions, and well as genes that differ among MPXV strains. Samples and controls included the infection study inoculum, MPXV Congo/Luc+ stock, wildtype MPXV (ROC 2003-358), and a primary lesion from RS15, which was expressing luciferase, tongue, liver, and blood

from RS14, and an oral swab from RS14 on day 19 post-infection. All DNA samples were extracted using Zymo® g-DNA kit (Zymo Research, Irvine, CA). Quickload Taq 2X Master mix or Phusion High fidelity DNA polymerase (New England BioLabs, Ipswich, MA) , following the manufacturer’s suggested protocol on a Geneamp PCR system 9700 (Applied Biosystems, Foster City, CA) . Some PCR products were cleaned using Zymo PCR purification kit (Zymo Research, Irvine, CA) and sequenced using Sanger sequencing at the University of Wisconsin Biotechnology Center (Madison, WI).

Finally, to estimate the concentration of wildtype versus recombinant virus, we performed real time PCR (as described in Materials and Methods, Quantitative Real Time PCR) to amplify a 150 bp fragment of the luciferase gene (PCR 10) and two fragments (PCR 11 and 12) that are present only in wildtype, non-recombinant virus surrounding the recombination site (S2 Table A). Using wildtype virus (MPXV/Congo) DNA, a standard curve was created for PCRs 11 and 12. DNA from another luciferase-containing virus, which was previously confirmed to only contain wildtype virus [20], was used to create a standard curve for PCR 10.

| PCR | **Genes Amplified** | **Product Size** | **Forward Primer** | **Reverse Primer** |
|---|---|---|---|---|
| PCR 1 | Luc+ cassette fragment | 312 | GAGCACGGAAAGACGATGAC | CCAATCTCCGGTCGCTAA |
| PCR 2 | Luc+ cassette fragment | 500 | GAGCACGGAAAGACGATGAC | TCGTTATTGATGACCTGGTGG |
| PCR 3 | 176-177 intergenic region | 2575/5154 | ATGGCACGATTGTCAATACTT | CTCGTCGTAATTGGGTTCCTC |
| PCR 4 | left and right flanks | 500/2851 | CAGGGCCGGCCGGACCGGACT<br>TACATAAATATCTGGG | GCGCCAGGCGCGCCGTTAAAA<br>TACATTCTAATACGG |
| PCR 5 | complement | 298 | TAAGTACATATGCCATTTTTT<br>GCTTTCTGTATCC | AAGATCGGCCGCACTGCCATT<br>GTTTTTGAGC |

| | | | | |
|---|---|---|---|---|
| PCR 6 | CCXR | 759 | TCAGACACATGCTTTGAGTTTTG | TAGAAACAATATATTGTCCTGGCA |
| PCR 7 | envelope | 1045 | ATGATGACACCAGAAAACGACG | TCTTTTAACGCATAGTACAGATTGA |
| PCR 8 | qPCR | 150 | GAGCACGGAAAGACGATGAC | CTTGCTCCACAAACACAACTCC |
| PCR 9 | qPCR | 150 | TAATCGTATTTGCGCGATGG | GACGAAGATTGGCCTCAACC |
| PCR 10 | qPCR | 150 | ACAGCTGTAAATACAGCGGC | CCCAAGTAATGCATTAGGTAAGT |

**Table A.** Primer sequences for PCRs used to determine why RS14, the sentinel rope squirrel, was infected with Monkeypox virus, but no luminescence was detected. Primer target sequences within and around the luc+ cassette from RS14, RS15 (an animal infected with a luminescent MPXV), and stocks from both wildtype MPXV/Congo and recombinant, luciferase expressing, MPXV/Congo/Luc+.

## Results

Luminescence was not detected in RS14 tongue or liver by plate luminometer 24 or 48 hours after infection. The fixed plate contained many viral plaques, confirming that virus was present and replicating in the cells without expression of luciferase. The positive control, MPXV Congo/Luc+, was luminescent, as expected.

The results of PCR 1 and 2, which amplify a 312 and 500 base pair fragment within the luciferase cassette, showed that RS14 oral swab, liver, and tongue, as well as wildtype MPXV/Congo, were negative for the cassette, while the MPXV/Congo/Luc+ was positive (Figure A). Interestingly, PCRs 3 and 4, which include the flanking regions outside of the recombinant site, show evidence of the presence of both wildtype and recombinant viruses in both RS15 lesion and MPXV/Congo/Luc+ stock, while RS14 tongue only had the wildtype band (Figures B-C).

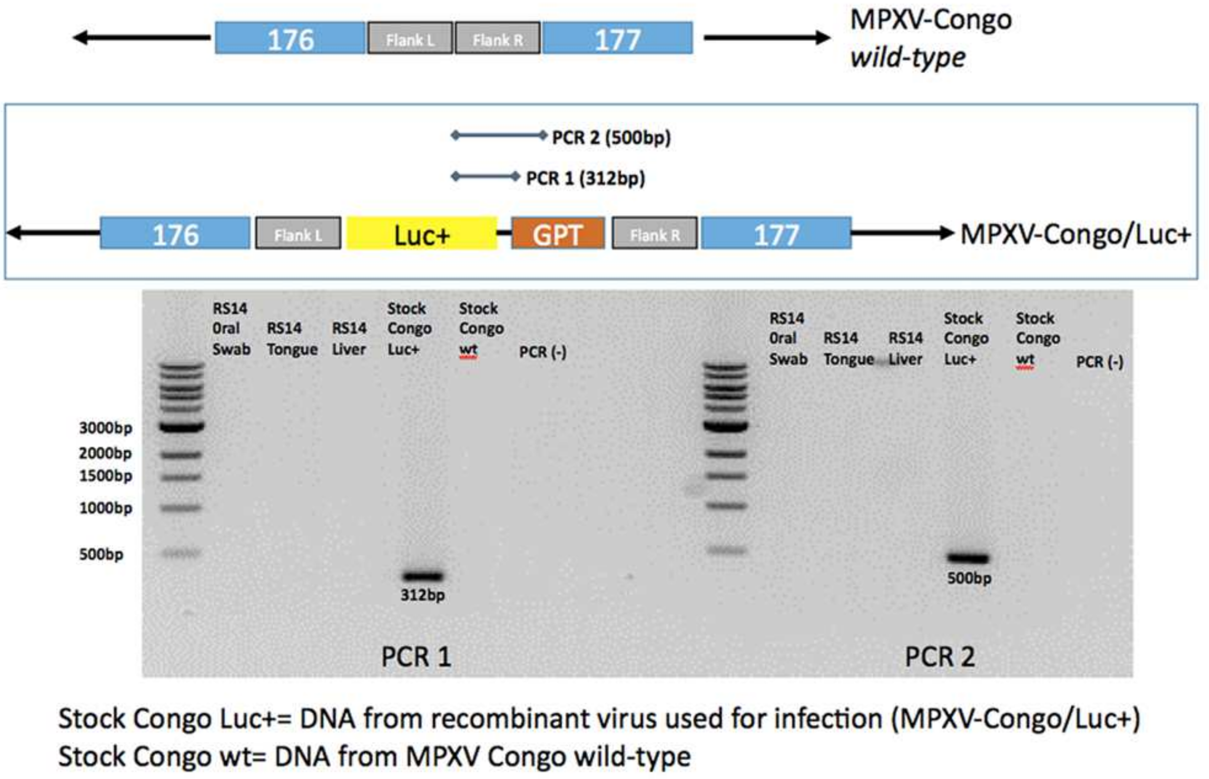


**Figure A.** PCR1 and PCR2 gel electrophoresis. The gene map above shows that PCR 1 targeted a 312 bp section in the luciferase cassette. Samples from RS14, including oral swab on day 18, tongue, and liver, show that the infecting virus did not contain the luciferase gene, but the control (MPXV/Congo/Luc+ stock) did. Likewise, in PCR 2, the MPXV/Congo/Luc+ stock was positive for a 500 bp section containing portions of the luciferase and GPT genes, while RS14 samples did not.

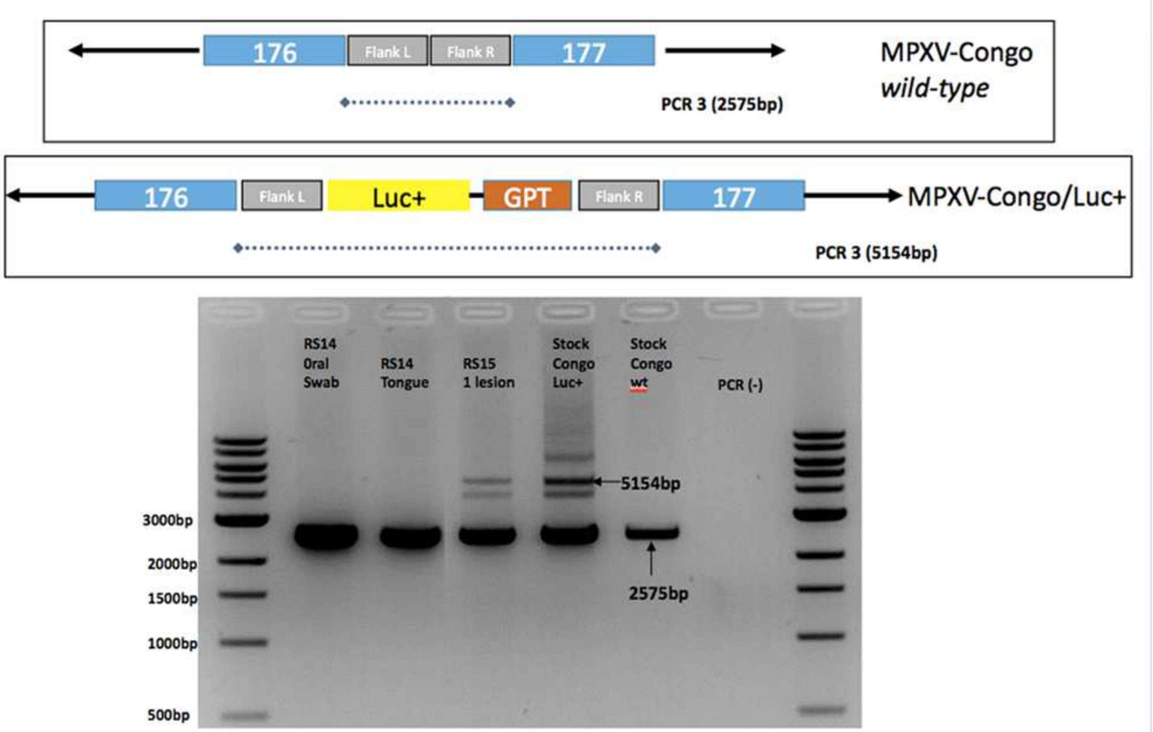


**Figure B.** As shown above, PCR 3 amplified the right and left flanking regions of where the Luc+ cassette was inserted in recombinant MPXV/Luc+. The amplicon is expected to be longer (5154 vs 2575 bp) in the recombinant MPXV with the luciferase/GPT cassette. RS15 and the MPXV/Congo/Luc+ stock, which was used to inoculate rope squirrels, was positive for both bands, while RS 14 tongue and RS14 oral swab were only positive for the wildtype, shorter amplicon.

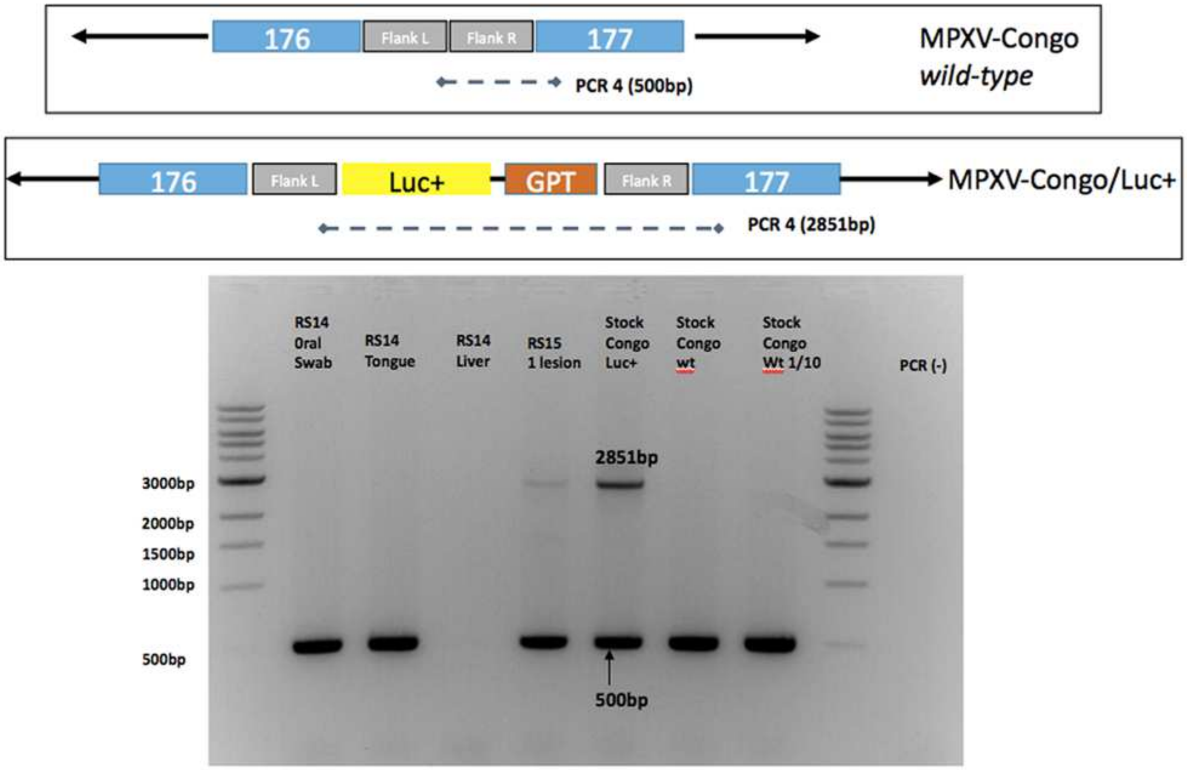


**Figure C.** PCR 4 amplifies a portion of the luciferase/GPT cassette from the left flanking region to the downstream gene, gene 177. In non-recombinant MPXV, the amplicon is 500 bp, while it is expected to be 2851 if the cassette is present. MPXV/Congo/Luc+ and RS15 skin lesion are positive for both bands, while samples from RS14, the sentinel rope squirrel, are only positive for the smaller, 500 bp band.

To evaluate that the non-recombinant MPXV was the same as the parental strain, and not a contaminant from another strain or potentially from a pre-existing infection in the squirrel, we performed three additional PCRs. PCR 5 amplified the complement gene, which is not present in West African origin MPXV strains. This showed that all RS14 sample viruses and a positive control of an MPXV isolate from Republic of Congo (ROC-2003-358) contained the complement gene and was central African in origin. The negative control, a West African origin strain from the US outbreak (USA-2003-044), did not amplify this gene. PCR 6 and 7 amplified

CCXR and the envelope gene, which are variable among central African strains of MPXV. These PCRs were positive and sequences of the amplicons were 100% identical to the parental MPXV strain (ROC-2003-358). Finally, the quantitative real time PCRs (PCRs 8, 9, 10) showed that the stock and the study inoculum contained approximately equal amounts of the wildtype and recombinant MPXV/Luc+ (1:1.13 recombinant: non-recombinant).

**Conclusions.** Analysis of the luciferase cassette in MPXV/Congo/Luc+ indicates that the inoculum and recombinant MPXV stock contained two populations of viruses, recombinant and non-recombinant. RS15 lesion also contained both wildtype and recombinant viruses, although luminescence was detected in the live animal. RS14 liver, tongue and oral swab contained mostly or entirely wildtype virus, but the blood showed evidence of the circulation of some recombinant virus. While all animals in this study were inoculated with both wildtype and recombinant viruses, both viruses are the same strain of MPXV, therefore pathology and virulence characteristics are expected to be the same, as shown in [29].
