# Supplementary material for: Characterization of Monkeypox virus infection in African rope squirrels (Funisciurus sp.)
Source: PLoS Negl Trop Dis. 2017 Aug 21;11(8):e0005809. doi: 10.1371/journal.pntd.0005809 (PMC5578676; doi:10.1371/journal.pntd.0005809)
Supplement: S2 Table — (PDF) [file pntd.0005809.s004.pdf]

Treatment	Animal ID	Days post-infection	Dorsal luminescence [p/s/cm²/sr]
sentinel	RS14	3	9.86E+02
sentinel	RS14	6	9.88E+02
sentinel	RS14	8	1.10E+03
sentinel	RS14	11	1.02E+03
sentinel	RS14	13	1.13E+03
ID	RS12	3	1.31E+04
ID	RS12	6	3.26E+05
ID	RS12	8	1.53E+04
ID	RS15	3	1.66E+03
ID	RS15	6	3.57E+05
ID	RS15	8	1.62E+05
ID	RS15	11	2.94E+05
ID	RS15	13	7.07E+05
ID	RS15	15	1.51E+04
ID	RS15	18	1.07E+03
ID	RS15	20	1.22E+03
ID	RS15	22	1.09E+03
ID	RS17	3	1.24E+04
ID	RS17	6	2.82E+04
ID	RS17	8	1.41E+04
ID	RS17	11	3.54E+03
ID	RS17	13	5.00E+03
ID	RS17	15	8.50E+02
ID	RS17	18	8.88E+02
ID	RS17	20	1.20E+03
ID	RS17	22	1.17E+03
ID	RS17	27	9.65E+02
ID	RS18	3	2.35E+05
ID	RS18	6	2.17E+05
ID	RS18	8	4.89E+04
ID	RS18	11	1.24E+04
ID	RS18	13	2.10E+03
ID	RS18	15	1.31E+03
ID	RS18	18	1.09E+03
ID	RS18	20	1.38E+03
ID	RS18	22	1.27E+03
ID	RS18	27	1.46E+03
IN	RS11	3	2.04E+03
IN	RS11	6	5.82E+03
IN	RS11	8	1.12E+04
IN	RS11	11	2.43E+04
IN	RS11	13	5.51E+04
IN	RS11	15	1.07E+03
IN	RS11	18	1.02E+03
IN	RS11	20	1.02E+03
IN	RS11	22	9.45E+02

IN	RS11	27	1.04E+03
IN	RS13	3	3.25E+03
IN	RS13	6	2.05E+04
IN	RS13	8	4.51E+04
IN	RS13	11	3.25E+05
IN	RS16	3	9.76E+02
IN	RS16	6	1.63E+03
IN	RS16	8	3.50E+04
IN	RS16	11	1.05E+06
IN	RS16	13	7.70E+05
IN	RS19	3	1.19E+03
IN	RS19	6	4.00E+03
IN	RS19	8	1.62E+04
IN	RS19	11	1.93E+05

Ventral luminescence [p/s/cm²/sr]
1.60E+03
1.55E+03
1.63E+03
1.79E+03
1.56E+03
1.90E+04
5.83E+05
71710
2.26E+03
2.33E+05
70590
1.49E+05
4.89E+05
9.24E+04
2.07E+03
1.96E+03
2.16E+03
2.00E+03
2.23E+03
4.95E+03
8.83E+03
8.15E+03
8.26E+02
1.58E+03
1.80E+03
1.40E+03
1.43E+03
1.16E+03
1.88E+04
6.40E+04
9.71E+03
2.81E+03
1.46E+03
1.46E+03
1.76E+03
1.68E+03
2.16E+03
1.50E+04
2.63E+04
8.13E+04
5.32E+04
4.79E+04
2.68E+03
1.11E+03
1.18E+03
1.27E+03

1.22E+03
4.09E+04
1.96E+06
7.77E+05
1.54E+06
1.68E+03
3.56E+03
1.98E+05
1.42E+06
6.27E+05
3.29E+03
4.33E+04
1.04E+05
1.81E+06
